# Lupeol Accumulation Correlates with Auxin in the Epidermis of Castor

**DOI:** 10.3390/molecules26102978

**Published:** 2021-05-17

**Authors:** Donghai Li, Cheng Pan, Jianjun Lu, Wajid Zaman, Huayan Zhao, Jixing Zhang, Shiyou Lü

**Affiliations:** 1 Key Laboratory of Plant Germplasm Enhancement and Specialty Agriculture, Wuhan Botanical Garden, Chinese Academy of Sciences, Wuhan 430074, China; lidonghai17@mails.ucas.edu.cn (D.L.); pancheng@wbgcas.cn (C.P.); lujianjun15@mails.ucas.ac.cn (J.L.); 2 University of Chinese Academy of Sciences, Beijing 100049, China; shangla123@gmail.com; 3 State Key Laboratory of Systematic and Evolutionary Botany, Institute of Botany, Chinese Academy of Sciences, Beijing 100093, China; 4 State Key Laboratory of Biocatalysis and Enzyme Engineering, School of Life Sciences, Hubei University, Wuhan 430062, China; huayanzhao@hubu.edu.cn; 5 College of Life Sciences and Food Engineering, Inner Mongolia University for Nationalities, Tongliao 028000, China; zhangjixing@imun.edu.cn

**Keywords:** castor, lupeol, transcriptome, auxin, terpene

## Abstract

Lupeol, a natural lupane-type pentacyclic triterpene, possesses various pharmacological properties, and its production attracts attention. Significant quantities of lupeol are deposited on the castor aerial organ surface and are easily extractable as a predominant wax constituent. Thus, castor might be considered as a potential bioreactor for the production of lupeol. The lupeol biosynthesis pathway is well known, but how it is regulated remains largely unknown. Among large numbers of castor cultivars, we targeted one accession line (337) with high levels of lupeol on its stem surface and low levels thereof on its hypocotyl surface, implicating that lupeol synthesis is differentially regulated in the two organs. To explore the underlying mechanisms, we did comparative transcriptome analysis of the first internode of 337 stem and the upper hypocotyl. Our results show that large amounts of auxin-related genes are differentially expressed in both parts, implying some possible interactions between auxin and lupeol production. We also found that several auxin-responsive *cis*-elements are present in promoter regions of *HMGR* and *LUS* genes encoding two key enzymes involved in lupeol production. Furthermore, auxin treatments apparently induced the expression levels of *RcHMGR* and *RcLUS.* Furthermore, we observed that auxin treatment significantly increased lupeol contents, whereas inhibiting auxin transport led to an opposite phenotype. Our study reveals some relationships between hormone activity and lupeol synthesis and might provide a promising way for improving lupeol yields in castor.

## 1. Introduction

Lupeol is a naturally occurring pentacyclic triterpene and has been studied for its interesting pharmacological properties, for instance as anti-cancer, anti-diabetes, and anti-inflammation agents [1]. Lupeol also serves as a precursor for the synthesis of lupane-type triterpenoids, such as betulinic acid (BA) and its derivatives, which have been shown to exert anticancer and anti-HIV effects [2]. Thus, the production of lupeol has raised some interest, and tremendous efforts have been made worldwide during the last three decades by worldwide researchers to test its clinical use and action mechanisms for the treatment of various disorders [3]. Lupeol is found in various vegetables, fruits, and medicinal plants, but the quantity of lupeol in these organs is limited, thus restricting its commercial use [1]. Therefore, it is necessary to explore new resources for generating lupeol as well as new ways to increase its yields.

Lupeol is mainly synthesized via the mevalonic acid (MVA) pathway, in which 11 enzymes are involved [4]. The MVA pathway begins with the condensation of two acetyl-CoA into acetoacetyl-CoA (AcAcCoA) catalyzed by acetoacetyl-CoA thiolase (AACT), AcAcCoA is then finally converted into isopentenyl diphosphate (IPP) through a series of enzymatic reactions in the cytosol, including 3-hydroxy-3-methylglutaryl-CoA synthase (HMGS), 3-hydroxy-3-methylglutaryl-CoA reductase (HMGR), mevalonate kinase (MK), 5-phosphomevalonate kinase (MVP), and 5-diphosphomevalonate decarboxylase (PMD) [5]. The C5 IPP are then subsequently converted into C15 farnesyl pyrophosphate (FPP) and C30 squalene mediated by FPP synthase (FPS) and squalene synthase (SQS), respectively. Squalene is then oxidized to 2,3-oxidosqualene by squalene monooxygenase (SQE). Finally, the 2,3-oxidosqualene is cyclized into lupeol by lupeol synthase (LUS), a member of oxidosqualene cyclases (OSC), resulting in the production of lupeol. In this type of reaction, 2,3-oxidosqualene is also cyclized into various triterpenoids by other OSCs, but LUS is only specific for lupeol production. Our previous work shows that there are in total 21 genes encoding the above-mentioned 11 enzymes in castor (*Ricinus communis* L.) [6]. In epidermis and stele, these genes showed various expression patterns, some genes are epidermal-specific, and some are expressed in both sections. HMGR catalyzing is a rate-limiting step in the MVA pathway and is encoded by three genes in castor, among which only one, *HMGR*, is epidermal-specific [5,6]. LUS is specifically responsible for lupeol synthesis and is encoded by two genes in castor, both of which are highly expressed in the epidermis [4,6,7]. Besides, other genes involved in the MVA pathway, including *HMGS*, *SQS,* and one of *SQEs*, are also highly expressed in the epidermis [6]. These genes showing epidermis-specific expression patterns are supposed to be closely related to the lupeol biosynthesis pathway. Besides the MVA pathway, the methylerythritol 4-phosphate (MEP) pathway might also provide substrates for synthesizing lupeol, although it only plays minor roles [6].

Castor accumulates high amounts of lupeol on its aerial surface, and as a predominant constituent, it crosslinks with other constituents, thereby forming thread-shape wax crystals [6,7]. Lupeol synthesis is well identified, but its regulation is still unclear. To find out possible regulation mechanisms, we selected one cultivar, 337, as a target, since lupeol distribution patterns varied greatly in hypocotyls and stems, in other words, the hypocotyl is glossy (also known as wax-deficient), whereas the stem is glaucous, indicating that lupeol synthesis is differentially regulated in the two organs. To explore the underlying mechanism, we performed a comparative transcriptome analysis of hypocotyl and the adjacent stem internode. Surprisingly, large amounts of auxin-related genes are differentially expressed in the hypocotyl and the adjacent stem internode, implying that auxin might regulate lupeol biosynthesis. We also found that auxin-responsive *cis*-elements are present in the promoter regions of *RcHMGR* and *RcLUS*, which are two key genes involved in lupeol synthesis. We further checked the responses of the two genes to auxin treatment and compared lupeol contents in leaves treated with auxin or auxin transport inhibitor. Our results proved that auxin acts as a positive regulator of lupeol biosynthesis.

## 2. Results and Discussion

### 2.1. Castor Accession Line 337 Has Less Lupeol Content on Its Hypocotyl Compared to the Stem

**Figure 1:**
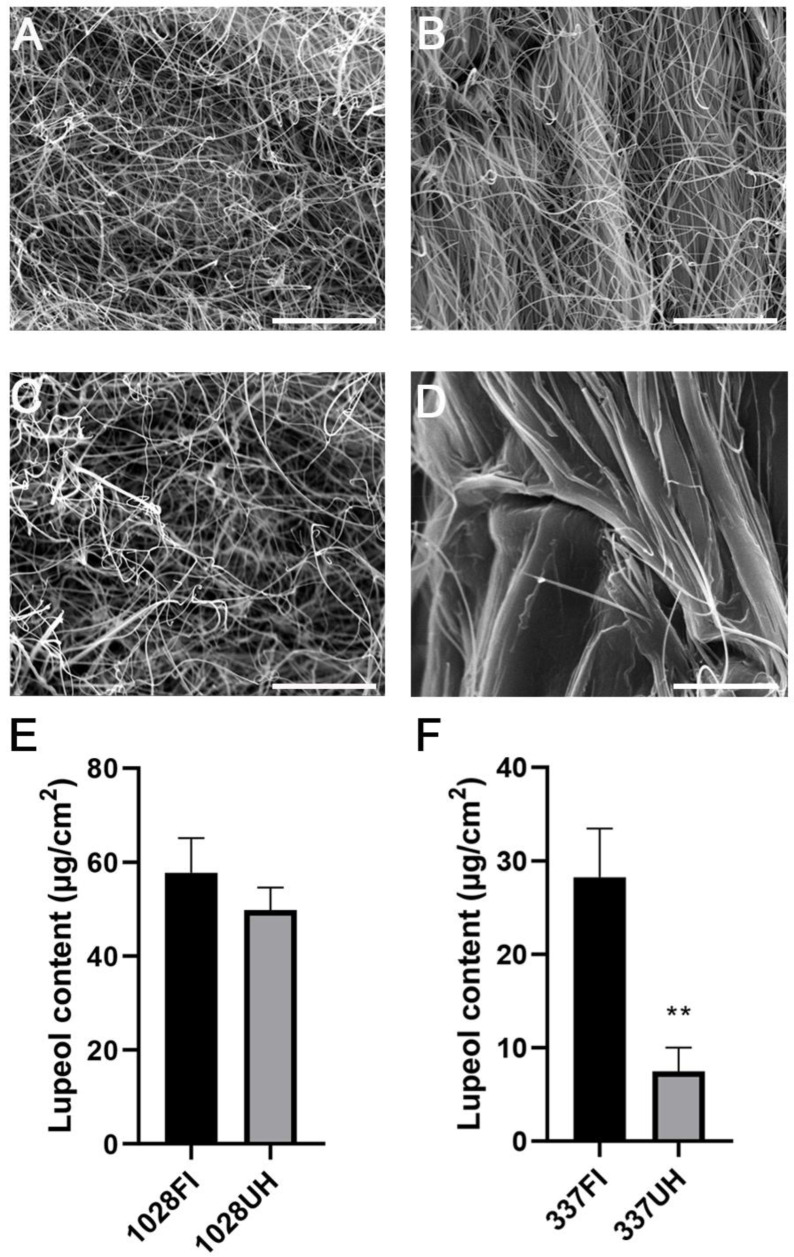
Wax phenotype of castor upper hypocotyl and first internode of castor accession lines 1028 and 337. SEM analysis was performed to check wax crystals coating on the first stem internode (FI) of 1028 (**A**) and 337 (**B**) as well as upper hypocotyl (UH) of 1028 (**C**) and 337 (**D**), respectively. Bar = 10 μm. (**E**,**F**) Lupeol content in the FI and UH of 1028 and 337. The black and grey boxes indicate the lupeol content of FI and UH accordingly. ** *p* < 0.01. Error bars represent ± SD (*n* = 3–4).

Lupeol was previously identified as a predominant wax component in the castor epidermis, and its content in total stem wax is correlated with the formation of epicuticular wax crystals on stems of glaucous individuals [7]. Our previous work indicated that the lupeol content varied significantly among different castor varieties [6]. Based on the glossy surface phenotype of either hypocotyls or stems of 4-week-old plants, we screened 395 castor individuals collected from different regions of China and Africa, previously described in earlier work [8], and identified 20 wax-deficient mutants (unpublished data). The surface of both hypocotyl and stem of most castor varieties is usually enclosed with large amounts of wax forming thread-like wax crystals, as shown in Figure 1A,C [6]. However, we noticed that one castor accession line 337 showed a unique wax-deficient phenotype, in other words, its hypocotyl is glossy, but its stem is glaucous, which is easily discernible.

To explore if the wax biosynthesis process is disturbed in upper hypocotyls, we observed the wax phenotype of the upper hypocotyls using scanning electron microscopy (SEM). Our results reveal that only a few epicuticular wax crystals are sparsely distributed on the upper hypocotyl. By contrast, large amounts of thread-like wax crystals were densely scattered on the first stem internode (Figure 1B,D). We also extracted cuticular waxes from upper hypocotyls and their adjacent stem internodes, respectively. The total wax loads in the upper hypocotyl were only 39% that of the first stem internode, in which lupeol as the predominant component of waxes is drastically decreased to 29% to that of the first internode (Figure 1F). β-Amyrin and alkanes are also more or less reduced, and other wax components are a little different between the two parts (Table S1, Supplementary Materials); whereas no significant differences were found in total wax loads, lupeol content, and other wax components between the first stem internode and the upper hypocotyl of accession line 1028 (Figure 1E, Table S1), which is consistent with the Scanning electron microscopy (SEM) results (Figure 1A,C). Therefore, 337 individuals were suitable material for investigating the regulation mechanism of lupeol synthesis.

### 2.2. RNA-Sequencing, Transcript Assembly, and Annotation

**Figure 2:**
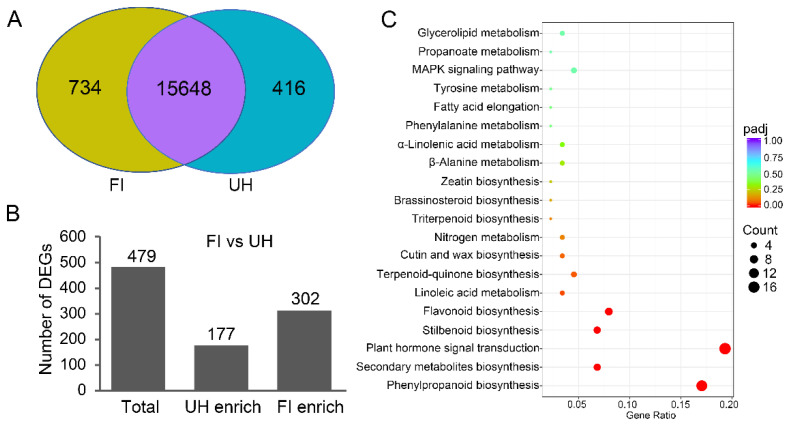
Transcriptome analysis identifies differentially expressed genes (DEGs) between upper hypocotyl (UH) and the first stem internode (FI) of the stem of accession line 337. (**A**) Venn diagram of detected genes in transcriptome of UH and FI. The regions filled with purple, yellow, and blue indicate genes detected in both organs, only in FI, and only in UH, respectively. (**B**) Differentially expressed genes between FI and UH. (**C**) Enrichment of Kyoto Encyclopedia of Genes and Genomes (KEGG) biochemical pathways in the FI.

To investigate the intrinsic molecular mechanism, we performed a comparative transcriptome analysis of hypocotyl and the first stem internode to explore why lupeol accumulation patterns are different in both parts. Total RNA was extracted from the upper hypocotyls and first internodes, respectively, of 4-week-old accession line 337 plants. Six cDNA libraries were constructed for further RNA-seq analysis. A total of 43.43 Gb clean data were generated, and 289, 539, and 324 clean reads were obtained. Q30 values of each sample ranged from 93.96% to 95%. All clean reads were mapped to the published reference genome of *Ricinus communis* L. [9] and the ratio of the mapped reads of each sample ranges from 92.87% to 94.96% (Table S2, Supplementary Materials). After assembly, a total of 16,798 genes were detected and successfully annotated in several public databases, including Clusters of Orthologous Groups of proteins (COG), Gene Ontology) GO, Kyoto Encyclopedia of Genes and Genomes (KEGG), Swiss-Prot, eggNOG, and NR (NCBI non-redundant protein sequence database) databases. A total 16,382 and 16,064 expressed genes were detected in the first stem internodes and upper hypocotyls, respectively, among which 734 genes were only found in the first stem internodes and 416 genes only in the upper hypocotyls (Figure 2A).

### 2.3. Comparative Transcriptome Enrichment Analysis between Upper Hypocotyl and First Internode of the Stem of Accession Line 337

Compared with those genes expressed in upper hypocotyl, a total of 479 differentially expressed genes (DEGs) were detected in the first stem internode with a glaucous phenotype, of which 302 genes were found upregulated and 177 genes were downregulated (Figure 2B, Tables S3 and S4, Supplementary Materials). To investigate which pathways are differentially regulated in the upper hypocotyl and the first stem internode, gene ontology (GO) assignments and a KEGG pathway enrichment analysis were performed on all DEGs. Furthermore, 31 GO terms and 6 KEGG pathways were significantly enriched (Tables S5 and S6, Supplementary Materials). Surprisingly, lupeol biosynthesis-related pathways were not significantly enriched. But interestingly, DEGs related to plant hormone were significantly enriched in either GO term “in response to auxin” or KEGG pathway “Plant hormone signal transduction” (Figure 2C, Table S5).

### 2.4. Genes Closely Related with Lupeol Biosynthesis in Castor Epidermis

Our previous work showed that a total of 21 genes encode 11 enzymes related to lupeol synthesis [6]. Most of these genes are not differentially regulated, but two genes, one *RcHMGR* (LOC8258747) and *RcLUS* (LOC8280320), were significantly upregulated in the first stem internode where lupeol are abundantly accumulated (Table S7, Supplementary Materials). We also checked their expression levels in the upper hypocotyl and the first stem internode by quantitative reverse transcription-polymerase chain reaction (qRT-PCR) to verify the authenticity of RNA-seq results. Consistent with RNA-seq results, the expression levels of *RcHMGR* and *RcLUS* in the first stem internodes by far exceeded that in the upper hypocotyls (Figure S1, Supplementary Materials). It is noteworthy to mention that mRNA of *RcHMGR* and *RcLUS* was found specifically enriched in the epidermal cells where lupeol is mainly synthesized [6], thus we hypothesized that both RcHMGR and RcLUS might represent key enzymes determining the shunting of acetyl-CoA pools to lupeol production. Besides the genes related to the MVA pathway, we also checked the expression levels of genes associated with the MEP pathway, though it only plays a subordinate role in lupeol synthesis (Table S8, Supplementary Materials) [6]. We found that only *1-deoxy-d-xylulose-5-phosphate synthase* is significantly increased in the first internode, suggesting that the MEP pathway might also contribute to lupeol production in castor (Table S8).

In addition, we found that several transporter genes are also highly expressed in the first internode containing high amounts of lupeol, encoding three *ABCG* transporter genes and one lipid transfer protein (*LTP*) (Table S3). ABCG proteins and LTPs are well identified to be involved in the transport of aliphatic wax constituents [10,11,12,13,14]. It is possible that lupeol transport might act in similar ways and the abundant accumulation of lupeol requires more transporters for its utmost secretion.

### 2.5. Auxin Signaling or Early Response Pathway Is Highly Enriched in the Upregulated DEGs

Interestingly, based on the GO analysis, we found that 22 DEGs were enriched in “response to auxin”, among which, 20 genes were upregulated and 2 genes are downregulated. KEGG analysis also revealed that 17 DEGs are related to “Plant hormone signal transduction pathway”, among which, 12 genes are related to auxin signal transduction with 10 genes falling into the upregulated group and 2 genes falling into the downregulated group (Tables S6 and S7). Combined with both GO assignment and KEGG enrichment analysis results, we identified that 28 out of the total 302 upregulated DEGs (about 9.3%) were related to the auxin response or auxin signaling pathway (Table 1), which include two *AUX/indole-3*-acetic acids (*AUX/IAAs*), one *Gretchen Hagen3* (*GH3*), five *auxin*-induced protein *15As* (*Aux15As*), and 20 small auxin-up RNAs (*SAURs*, five *SAUR21*-like genes, one *SAUR 62*-like gene, two *SAUR63*-like genes, and *12 SAUR68*-like genes) (Table 1). qRT-PCR was also performed to verify the reliability of RNA-seq results (Figure S1). *AUX/IAA*s, *GH3*s, and *SAUR*s are known to represent the three largest gene families that are involved in early auxin-response [15,16,17]. The expression of these genes is usually induced by exogenous auxin treatment or endogenous auxin accumulation. Interestingly, among all the up-regulated *SAURs*, 15 *SAURs* were closely related to *Arabidopsis SAUR63* subfamily genes [18]. *Arabidopsis SAUR63* subfamily proteins share over 69% amino acid sequence identity sharing functional redundancy with each other [19,20]. Notably, AtSAUR63 was reported to positively regulate auxin transport [19]. Even though the functions of *SAUR63* subfamily genes in castor are still unclear, the proteins coded for display similar functions to AtSAUR63. Therefore, the upregulation of *SAUR63*-like genes might also promote auxin transport in the first internode of accession line 337 stems, thus triggering auxin signaling or early response.

To date, the regulatory interaction between auxin and lupeol synthesis is not reported yet. However, several studies suggested that auxin promotes the biosynthesis of some secondary metabolites including sesquiterpenes in *Lippia dulcis* [21], indole alkaloids in *Amsonia elliptica* [22], and ginsenoside in *Panax* Hybrid [23], possibly by activating the MVA pathway [24]. In our study we found that the auxin-related genes are highly expressed in the first internode of accession line 337 stems where lupeol is highly accumulated, implicating that a potential interaction might be present between auxin and lupeol biosynthesis in castor.

### 2.6. Auxin Treatment Enhanced Lupeol Accumulation in the Epidermis of Castor Leaves

To confirm that auxin is indeed related to lupeol synthesis, we first analyzed the 2 kb upstream region of both *RcHMGR* and *RcLUS* using PlantCARE (http://bioinformatics.psb.ugent.be/webtools/plantcare/html/, accessed on 17 May 2021) [25] since the two genes are highly expressed in the first stem internode. Our results revealed that two auxin-response elements, AuxRE (GAGACA, −1539 to −1533) and TGA (AACGAC, −1582 to −1577), were in the *cis* strand of the promoter region of *RcHMGR* and one auxin-response element TGA (AACGAC, −776 to −771) is present in the anti-strand of the promoter region of *RcLUS* (Figure S2), implying that the expression of *RcHMGR* and *RcLUS* might be induced by auxin. To verify this hypothesis, we had chosen accession line 1028 instead of 337 for auxin treatments since the lupeol distribution of line 337 displays organ-specific wax deficiency phenotype, indicating the lupeol synthesis process is obviously disturbed in some organs of this line such as hypocotyls and leaves.

**Figure 3:**
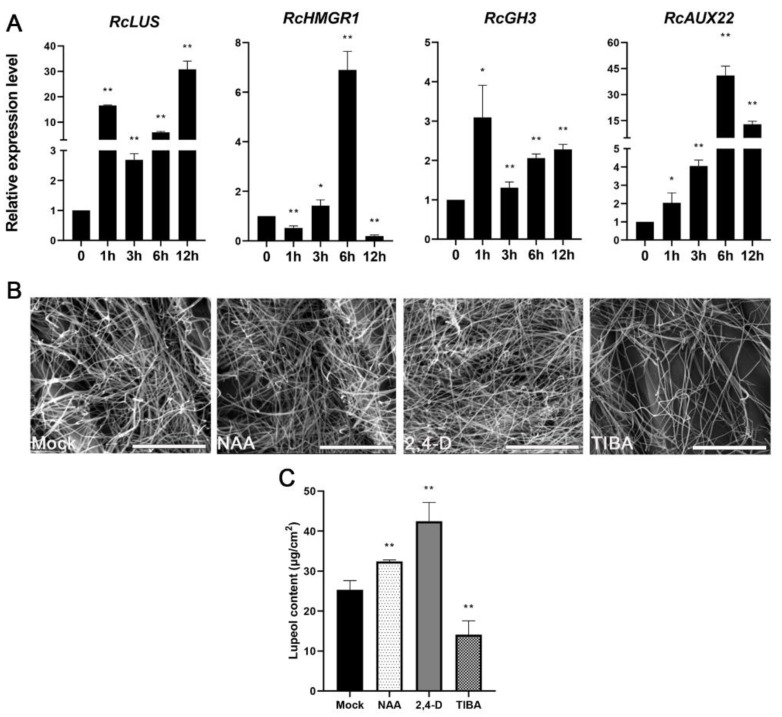
Lupeol levels are induced by auxin. (**A**) Relative expression levels of four different genes including *RcLUS* (LOC8280320), *RcHMGR* (LOC8258747), *RcGH3* (LOC8285710), and *RcAUX22* (LOC8271341) in the leaf of castor 1028 individual subjected to mock, 30 μM NAA for 0 h, 1 h, 3 h, 6 h, and 12 h. (**B**,**C**) Wax crystals (**B**) and lupeol content (**C**) on the surface of the third leaves of castor treated with mock, 30 μM NAA, 10 μM 2,4-D, and 30 μM TIBA, respectively. Bar = 10 μm. * *p* < 0.05, ** *p* < 0.01.

We firstly treated the leaves of accession line 1028 with naphthaleneacetic acid (NAA) or mock and then checked whether *RcHMGR* and *RcLUS* are responsive to auxin. As shown in Figure 3A, both genes could be significantly upregulated by auxin. To further prove that lupeol yields are indeed closely related to auxin levels indeed, we also checked wax components in the third leaves of 2-week-old seedlings of 1028 that were treated with auxin (NAA, 2,4-D) and auxin transport inhibitor TIBA for two weeks. Auxin significantly increased lupeol levels while TIBA apparently inhibited lupeol accumulation (Figure 3C). Furthermore, we checked the wax crystals on these treated samples using SEM. Consistent with wax analysis results, NAA and 2,4-D-treated leaves produced more wax crystals than mock-treated leaves, whereas TIBA-treated leaves generated fewer wax crystals (Figure 3B) than mock-treated leaves. These results indicated that auxin signal transduction indeed promotes lupeol production in the castor epidermis, possibly by activating the expression of *RcHMGR* and *RcLUS*. Taken together, auxin might function as a positive elicitor for lupeol production in the castor epidermis.

In conclusion, with the aid of comparative transcriptome analysis, we found some possible relationships between auxin and lupeol biosynthesis in castor which is also experimentally proved in our study. Our study should be helpful to advance our understanding of how lupeol production in castor epidermis is regulated and might provide a clue for enhancing castor lupeol production in the field, for instance by spraying auxin-mimic chemicals in the future.

## 3. Materials and Methods

### 3.1. Plant Materials

Castor accession lines (No. 1028 and No. 337), previously reported in the study of Fan et al. [8], were obtained from the oil crop research institute, Chinese Academy of Agricultural Sciences. The two seed lines were germinated in water, then grown in pots filled with vermiculite only at 22 °C with a 16 h/8 h light/dark cycle and complete nutrients were periodically supplied. For transcriptomic analysis, total RNA was extracted from upper hypocotyls and first stem internodes, respectively, of 4-week-old accession line 337. To check gene responses to auxin treatment, we sprayed distilled deionized water (DDW) and 30 μM NAA, respectively, on the adaxial surface of the first pair of true leaves of two-week-old seedlings of 1028 individual and leaves were collected at 0, 1, 3, 6, and 12 h for RNA extraction. For wax analysis, we either sprayed distilled deionized water (DDW), 30 μM NAA, 10 μM 2,4-dichlorophenoxyacetic acid (2,4-D), and 30 μM 2,3,5-triiodobenzoic acid (TIBA) on the adaxial surface of two-week-old leaves once a day, for 14 days, and then the third leaves were harvested for wax chemistry analysis.

### 3.2. Cuticular Wax Analysis

Wax chemistry analysis was performed as previously described by Guhling et al. [7] with some modifications. Materials were immersed in chloroform (CHCl_3_) with *n*-tetracosane as internal standard. The solvent was then evaporated under a gentle stream of N_2_ while heating to 60 °C. Dried samples were derivatized by bis-N, N-(trimethylsilyl) trifluoroacetamide (BSTFA) in pyridine (30 min at 70 °C). Finally, the derivatized mixtures were redissolved in 100 μL CHCl_3_ and further analyzed by gas chromatography (GC) exactly as described by Liu et al. [6]. The quantification of different wax constituents was performed against the internal standard by manually integrating flame ionization detector (FID) peak areas.

### 3.3. Scanning Electron Microscopy (SEM)

Upper hypocotyls and first stem internodes of lines 337 and 1028 were dried at room temperature. The samples were then fixed on a tray by double-sided adhesive tape, coated with gold particles in a vacuum for two minutes (EM ACE200, Lecia, Wetzlar, Germany), and finally observed by SEM (Quanta250, FEI, Brno, Czech Republic).

### 3.4. RNA Extraction, cDNA Library Preparation, and Transcriptome Sequencing

Total RNA was extracted with Trizol reagent (Invitrogen, Carlsbad, CA, USA). RNA-seq were performed by Beijing Novogene Biological Information Technology Co., Ltd. (Beijing, China) RNA concentration was measured with Qubit RNA Assay Kit in Qubit 2.0 Fluorometer (Life Technologies, Carlsbad, CA, USA) and RNA integrity was assessed by the RNA Nano 6000 Assay Kit of the Bioanalyzer 2100 system (Agilent Technologies, Santa Clara, CA, USA). Sequencing cDNA libraries were generated with 3 μg RNA per sample using NEBNext UltraTM RNA Library Prep Kit for Illumina (New England Biolabs, Ipswich, MA, USA) and index codes were added for attributing sequences to each sample. After clustering index-coded samples, the generated cDNA libraries were sequenced on an Illumina Hiseq platform and 125 bp/150 bp paired-end reads were generated.

### 3.5. Transcriptome Assembly, Functional Annotation, and DEG Analysis

Raw data (raw reads) of fastq format were first processed through in-house Perl scripts. Clean reads were obtained by removing reads containing adapter, ploy-N, and low quality reads from raw data. Meanwhile, Q20, Q30, and GC content of the clean data were calculated. An index of the reference genome was built by Hisat2 (v2.0.5) and paired-end clean reads were aligned to the reference genome [9]. FeatureCountsv1.5.0-p3 was used to count the reads numbers mapped to each gene. Then fragments per kilobase of transcript per million fragments mapped (FPKM) of each gene were calculated based on the length of the gene and reads count mapped to this gene. Gene functional annotation was performed by blasting against the following databases: Clusters of Orthologous Groups of proteins (COG), Gene Ontology database (GO), Kyoto Encyclopedia of Genes and Genomes (KEGG), Swiss-Prot (A manually annotated, non-redundant protein sequence database), eggNOG (A database of orthology relationships, functional annotation, and gene evolutionary histories) and NR (NCBI non-redundant protein sequence database).

Differential expression analysis of two conditions/groups (two biological replicates per condition) was performed by the DESeq2 R package (1.16.1) with a significant threshold of >2 or <−2 [26]. Genes with adjusted *P*-value < 0.05 found by DESeq2 were assigned as differentially expressed genes (DEGs). Gene Ontology (GO) enrichment analysis of DEGs was implemented by the clusterProfiler R package, in which gene length bias was corrected [27]. The statistical enrichment of DEGs in KEGG pathways was tested by clusterProfiler R package [28].

### 3.6. Quantitative Reverse Transcription-PCR (qRT-PCR)

Total RNA isolation and first-strand cDNA synthesis was performed, respectively, by RNA extracting kit (Cat^#^ RP3302, Bioteke, Beijing, China) and HiScript III 1st Strand cDNA Synthesis Kit (Code: R312-02, Vazyme, Nanjing, China) according to the manufacturers’ instructions. qRT-PCR was performed with SuperReal PremMix Plus (SYBR Green, Cat^#^ FP205-02, TIANGEN, Beijing, China) in CFX Connect TM Real-Time System (B3491, BIO-RAD, Hercules, CA, USA). Three biological replicates were performed to evaluate the transcript abundance of each gene. Specific primers used for qRT-PCR of each tested gene are listed in Table S9. The relative expression level of each gene was calculated based on the 2^–ΔΔC^ algorithm using RcACT7 as internal control [29].

## Figures and Tables

**Table 1:** Genes known to be involved in auxin signaling or earlier auxin response are enriched in the first internode of stem compared to upper hypocotyl of accession line 337.

Gene-ID	log2FC *	*p*-Value	Gene Description
LOC8280179	2.297	0.0002	auxin-responsive protein IAA7
LOC8271341	1.957	0.0005	auxin-induced protein AUX22
LOC8285710	2.956	0.0002	indole-3-acetic acid-amido synthetase GH3.17
LOC107262116	3.171	0.0001	auxin-induced protein 15A-like
LOC107260799	4.896	0.0001	auxin-induced protein 15A-like
LOC107262113	6.189	0.0002	auxin-induced protein 15A-like
LOC107262111	3.754	0.0002	auxin-induced protein 15A-like
LOC107262117	5.028	0.0008	auxin-induced protein 15A-like
LOC107261577	4.484	0.0001	auxin-responsive protein SAUR21-like
LOC107261578	6.831	0.0000	auxin-responsive protein SAUR21-like
LOC107261581	7.976	0.0000	auxin-responsive protein SAUR21-like
LOC107261586	8.247	0.0000	auxin-responsive protein SAUR21-like
LOC107261587	7.936	0.0000	auxin-responsive protein SAUR21-like
LOC8283211	3.697	0.0003	auxin-responsive protein SAUR62-like
LOC8283214	7.806	0.0000	auxin-responsive protein SAUR63-like
LOC8283212	6.678	0.0000	auxin-responsive protein SAUR63-like
LOC8266878	8.459	0.0000	auxin-responsive protein SAUR68-like
LOC8266881	8.433	0.0000	auxin-responsive protein SAUR68-like
LOC8266894	8.010	0.0000	auxin-responsive protein SAUR68-like
LOC8266888	2.562	0.0003	auxin-responsive protein SAUR68-like
LOC8266882	6.531	0.0000	auxin-responsive protein SAUR68-like
LOC8266895	7.277	0.0000	auxin-responsive protein SAUR68-like
LOC8266875	6.060	0.0000	auxin-responsive protein SAUR68-like
LOC8266880	4.946	0.0004	auxin-responsive protein SAUR68-like
LOC8266887	6.710	0.0000	auxin-responsive protein SAUR68-like
LOC8283213	5.393	0.0003	auxin-responsive protein SAUR68-like
LOC8266876	6.118	0.0003	auxin-responsive protein SAUR68-like
LOC8266884	5.706	0.0008	auxin-responsive protein SAUR68-like

* FC = fold change (First Internode vs. Upper Hypocotyl).

## Data Availability

Data are available from the authors on request.

## Supplementary Materials

The following are available online, Figure S1: qRT-PCR validation of transcriptome data; Figure S2: Schematic representation of the auxin response cis-elements in a 2-kb sequence upstream of the start codon of *RcLUS* and *RcHMGR*, respectively, Table S1: Cuticular wax composition of both upper hypocotyls (UH) and first internodes (FI) of the stem of 337 and 1028 accession lines, Table S2: Valid data statistics of RNA sequencing, Table S3: Upregulated genes in the first internode of stem compared to upper hypocotyl of 337 individual, Table S4: Downregulated genes in the first internode of stem compared to upper hypocotyl of 337 individual, Table S5: GO categories significantly enriched in the first internode of the stem of 337 individual. The results are grouped into the molecular function (MF) and biological process (BP), Table S6: Enriched KEGG biochemical pathways in the first internode of the stem of 337 individual, Table S7: List of candidate genes of lupeol biosynthesis related to MVA pathway in castor. FC means fold change (first internode vs. upper hypocotyl), Table S8: List of candidate genes of lupeol biosynthesis related to methylerythritol 4-phosphate (MEP) pathway in castor. FC means fold change (first internode vs. upper hypocotyl), Table S9: Primers used in this study.
