# Supplementary material for: Lupeol Accumulation Correlates with Auxin in the Epidermis of Castor
Source: Molecules. 2021 May 17;26(10):2978. doi: 10.3390/molecules26102978 (PMC8156332; doi:10.3390/molecules26102978)
Supplement: Supplementary file 1 [file molecules-26-02978-s001.zip › Supplemental figures and Tables/Table S3.pdf]

Table S3. Upregulated genes in the first internode of stem compared to upper hypocotyl of 337 individual.

Gene-ID	log2FoldChange	pvalue	padj	Gene description
LOC8281236	8.859583418	3.41E-118	6.97E-114	non-specific lipid-transfer protein 1
LOC8263715	1.987269191	7.21E-08	2.04E-05	glycine-rich cell wall structural protein 1.8
LOC8285157	4.405824381	3.39E-10	2.03E-07	proline-rich protein 4
LOC8267002	1.642752017	3.03E-07	6.88E-05	glucomannan 4-beta-mannosyltransferase 2
LOC8288718	1.199339698	0.000989802	0.042331628	phosphoenolpyruvate carboxykinase (ATP)%2C transcript variant X2
LOC8278573	2.647542595	7.86E-08	2.20E-05	uncharacterized LOC8278573
LOC8270992	1.20177562	0.000359069	0.020817816	amino acid permease 3%2C transcript variant X1
LOC8273057	2.671779027	2.95E-05	0.003104204	xyloglucan endotransglucosylase/hydrolase 1
LOC8289094	3.228149802	1.70E-10	1.15E-07	glycine-rich cell wall structural protein 1.8
LOC8274394	2.174968534	6.10E-06	0.000882966	linoleate 13S-lipoxygenase 2-1%2C chloroplastic
LOC8288174	1.148964372	0.000111386	0.008610453	abscisate beta-glucosyltransferase
LOC8280320	2.877440334	0.000125996	0.009356628	lupeol synthase%2C transcript variant X1
LOC8280754	4.419160247	0.000449407	0.024072193	alpha-copaene synthase%2C transcript variant X1
LOC8280179	2.29691683	0.000221668	0.014453019	auxin-responsive protein IAA7
LOC8286448	2.582149348	2.30E-16	6.70E-13	vacuolar cation/proton exchanger 3
LOC8281915	2.186822586	1.34E-10	9.46E-08	MLP-like protein 423
LOC8258747	6.167913341	1.14E-33	1.16E-29	3-hydroxy-3-methylglutaryl-coenzyme A reductase 3
LOC8274409	3.376262062	6.95E-08	2.03E-05	DNA damage-repair/toleration protein DRT100
LOC8259431	3.210217991	5.31E-05	0.004911492	14 kDa proline-rich protein DC2.15
LOC8265755	4.933260271	9.46E-08	2.54E-05	uncharacterized LOC8265755
LOC8270019	1.472754056	0.001100482	0.045627152	heavy metal-associated isoprenylated plant protein 39%2C transcript variant X1
LOC8288283	1.223383603	0.000168783	0.011756067	mechanosensitive ion channel protein 6
LOC8286845	1.756130745	3.68E-08	1.17E-05	two-pore potassium channel 1%2C transcript variant X1
LOC8273429	2.830937573	1.96E-13	2.66E-10	sulfite exporter TauE/SafE family protein 3

LOC8258485	2.219034446	4.30E-08	1.35E-05	MADS-box protein SOC1%2C transcript variant X3
LOC8263153	1.778864325	1.04E-07	2.73E-05	beta-galactosidase 8
LOC8261035	0.970499676	7.59E-05	0.006505181	cytochrome P450 90A1
LOC8288996	1.26138181	0.000854729	0.037837987	probable boron transporter 2
LOC8287457	1.712046293	6.34E-11	5.31E-08	putative cell division cycle ATPase
LOC8287425	1.124754649	5.96E-05	0.005336436	probable polygalacturonase
LOC8270993	1.873353109	5.67E-05	0.005161284	amino acid permease 3
LOC8265756	2.884451255	2.08E-07	4.95E-05	tuberculostearic acid methyltransferase UfaA1%2C transcript variant X4
LOC8279153	10.19381947	1.97E-27	1.34E-23	expansin-A8
LOC8287121	1.425529842	0.000115836	0.008820806	uncharacterized LOC8287121
LOC8280192	6.251726801	1.83E-09	9.35E-07	3-ketoacyl-CoA synthase 11
LOC107261016	1.152118474	0.000317141	0.018922053	uncharacterized LOC107261016
LOC8272272	1.206037477	0.000702324	0.032976057	probable beta-D-xylosidase 5%2C transcript variant X2
LOC8287056	2.133409599	1.75E-12	1.98E-09	transcription factor BHLH089%2C transcript variant X2
LOC8286437	0.978261975	0.000551431	0.027994046	UDP-glycosyltransferase 71K1%2C transcript variant X1
LOC8268051	2.749241331	0.000230875	0.01486342	uncharacterized LOC8268051
LOC8264174	1.406517935	0.000637058	0.030881414	probable receptor-like protein kinase At2g39360
LOC8264121	3.355733685	2.20E-08	8.01E-06	potassium channel KAT1%2C transcript variant X2
LOC107262024	2.34703378	2.42E-08	8.50E-06	glycine-rich cell wall structural protein
LOC8279681	2.536512583	5.64E-05	0.005157854	UDP-glycosyltransferase 74E2
LOC8271341	1.957104671	0.000506764	0.026344083	auxin-induced protein AUX22
LOC8266552	4.858620708	2.61E-10	1.66E-07	probable 3-hydroxyisobutyryl-CoA hydrolase 2
LOC8279864	1.129417641	4.90E-05	0.004589432	thylakoid lumenal protein TL20.3%2C chloroplastic
LOC8283712	1.059146328	0.000786723	0.035758212	uncharacterized calcium-binding protein At1g02270%2C transcript variant X2
LOC8272286	3.529956929	1.60E-06	0.000290002	vacuolar iron transporter 1
LOC8278596	1.935240877	4.11E-06	0.000633766	strigolactone esterase RMS3
LOC8261626	6.298455759	1.28E-10	9.32E-08	expansin-A15%2C transcript variant X1

LOC8289465	2.909085818	4.80E-08	1.48E-05	caffeoyl-CoA O-methyltransferase
LOC8288921	0.986298729	0.00016151	0.011444793	phosphatidylinositol transfer protein 3%2C transcript variant X1
LOC8267425	3.928369241	3.43E-06	0.000558606	probable terpene synthase 13%2C transcript variant X1
LOC8261920	2.215477841	6.65E-10	3.88E-07	cyprosin%2C transcript variant X2
LOC8276155	2.533340523	3.44E-12	3.70E-09	two-pore potassium channel 1
LOC8283401	1.301564159	3.02E-05	0.003130137	protein LNK3
LOC8283269	1.280726954	0.000221489	0.014453019	transcription factor HRS1
LOC8287876	1.632539591	0.000261651	0.016329578	suppressor of disruption of TFIIS
LOC8269188	2.149515716	0.000120865	0.009135634	ACT domain-containing protein ACR4%2C transcript variant X2
LOC8289192	2.794534572	2.52E-09	1.20E-06	vicianin hydrolase%2C transcript variant X1
LOC8266677	3.593895064	5.05E-18	2.06E-14	protein ECERIFERUM 1
LOC8276154	2.869838135	4.11E-13	4.93E-10	two-pore potassium channel 1%2C transcript variant X1
LOC8278794	4.024355796	1.34E-14	2.73E-11	uncharacterized LOC8278794
LOC8273523	1.044662162	0.000404294	0.0226671	serine/threonine-protein kinase SAPK1
LOC8266310	2.301336416	0.001135444	0.046624027	protein IQ-DOMAIN 1
LOC8260657	2.054548058	9.26E-06	0.001234661	MLO-like protein 4%2C transcript variant X2
LOC8274963	1.115259716	0.000816551	0.036478895	aspartic proteinase
LOC8282947	1.347858916	4.26E-05	0.004099221	U-box domain-containing protein 15
LOC8286422	1.303958011	3.57E-05	0.003521081	MLP-like protein 328
LOC8258591	2.220476803	0.000620036	0.030344575	LRR receptor-like serine/threonine-protein kinase ERECTA
LOC8269705	3.586346365	1.07E-13	1.56E-10	aluminum-activated malate transporter 9%2C transcript variant X1
LOC8265217	2.006401618	1.97E-05	0.00229683	glutamate receptor 2.7
LOC8273482	1.424419413	0.000171641	0.011893193	uncharacterized LOC8273482
LOC8277118	1.412801617	0.000377962	0.021666999	BAHD acyltransferase DCR
LOC8284230	3.20630172	8.29E-07	0.000167602	cytochrome P450 77A1
LOC8259022	2.684861481	1.47E-07	3.76E-05	pectinesterase

LOC8263008	2.754547293	3.53E-09	1.60E-06	probable glycerol-3-phosphate acyltransferase 3
LOC8271380	2.612417178	8.89E-06	0.001217277	uncharacterized LOC8271380
LOC8277520	1.74028999	5.54E-05	0.005088765	pathogenesis-related protein PR-1
LOC8258352	1.066513988	0.000936294	0.040455998	probable inorganic phosphate transporter 1-7%2C transcript variant X2
LOC8284533	1.177953155	0.000607272	0.029935303	V-type proton ATPase subunit G1
LOC8279682	4.354007399	1.94E-07	4.66E-05	UDP-glycosyltransferase 74E2
LOC8274318	2.477093682	0.000624507	0.030456223	ABC transporter G family member 13
LOC8273266	11.06437898	2.08E-17	7.07E-14	ricin-like%2C transcript variant X1
LOC8281866	1.424905658	0.001080048	0.045074898	cyclic dof factor 3
LOC8274087	4.063475057	6.51E-11	5.31E-08	flavonol synthase/flavanone 3-hydroxylase
LOC8262905	2.076124376	0.000429253	0.023200223	uncharacterized LOC8262905
LOC8288961	1.640909443	0.000286432	0.017606922	glucose-1-phosphate adenylyltransferase large subunit 1
LOC8261776	2.64478762	0.000162074	0.011445016	amino acid permease 4
LOC8276338	1.996416028	0.001072242	0.04484082	CO(2)-response secreted protease
LOC8262635	1.57503256	0.000401047	0.022666059	uncharacterized LOC8262635
LOC8283500	4.032355013	2.85E-08	9.54E-06	3-ketoacyl-CoA synthase 2
LOC8270023	2.535691919	0.000156724	0.011222558	trichohyalin
LOC8282062	1.325020041	0.000136561	0.010024985	-
LOC8280577	2.881186224	0.000303959	0.018275028	gibberellin-regulated protein 14
LOC8265831	2.367291636	4.00E-09	1.74E-06	squamosa promoter-binding-like protein 8
LOC8278600	3.515143038	9.25E-10	5.25E-07	protein SODIUM POTASSIUM ROOT DEFECTIVE 2
LOC8259547	4.560575302	3.26E-11	3.02E-08	expansin-A4
LOC8268754	2.415805048	1.50E-06	0.000277342	probable acyl-activating enzyme 1%2C peroxisomal%2C transcript variant X1
LOC8274780	4.299054086	2.37E-10	1.56E-07	protein RICE SALT SENSITIVE 3
LOC8269206	1.256467543	0.000505306	0.026344083	squamosa promoter-binding-like protein 13A%2C transcript variant X4
LOC107262416	2.314160141	0.000430956	0.023200223	uncharacterized LOC107262416%2C transcript variant X2

LOC8279968	2.272810149	2.78E-07	6.44E-05	calcium-binding protein KRP1
LOC8287395	1.830882933	0.000996211	0.042444012	cytochrome P450 86A22
LOC8259873	1.871297467	0.000149263	0.010801955	caffeoylshikimate esterase%2C transcript variant X2
LOC8270682	1.534976657	0.000760661	0.034962989	CRAL-TRIO domain-containing protein YKL091C
LOC8282706	1.998765208	0.00070289	0.032976057	uncharacterized LOC8282706
LOC8285508	1.728939529	4.74E-05	0.004476216	sugar transporter ERD6-like 5%2C transcript variant X1
LOC8288853	1.496115092	0.001242354	0.049454058	uncharacterized LOC8288853
LOC8264513	1.632757532	7.71E-07	0.000157402	phosphate transporter PHO1 homolog 1
LOC8264231	3.901126576	4.11E-05	0.003979652	basic leucine zipper 61
LOC8268549	3.949159893	0.000455555	0.024337608	-
LOC8289602	1.735442343	1.85E-05	0.002242126	malonyl-CoA:anthocyanidin 5-O-glucoside-6"-O-malonyltransferase
LOC8288563	3.32921619	0.000791818	0.035791074	uncharacterized LOC8288563%2C transcript variant X1
LOC8265539	3.417746577	1.93E-05	0.00229683	secoisolariciresinol dehydrogenase
LOC8281202	1.932432426	0.000749345	0.034598731	NAD(P)H-quinone oxidoreductase subunit U%2C chloroplastic
LOC8261659	1.815797042	8.21E-06	0.001132314	uncharacterized LOC8261659
LOC8269110	5.200045903	1.39E-05	0.001763814	fasciclin-like arabinogalactan protein 6
LOC8278922	1.91807064	0.000343613	0.020092979	plant UBX domain-containing protein 10
LOC8277363	2.534910641	6.74E-07	0.000141829	mitochondrial outer membrane protein porin of 36 kDa
LOC8267409	3.305088011	0.000105544	0.008284378	sulfite exporter TauE/SafE family protein 2%2C transcript variant X1
LOC8263576	1.877138773	0.000430318	0.023200223	GDSL esterase/lipase 4
LOC8284294	1.524647586	0.001212087	0.048789477	cytochrome P450 89A2
LOC8284177	7.563004865	1.78E-19	9.08E-16	glycine-rich protein 5
LOC8288834	4.333019407	1.96E-05	0.00229683	uncharacterized LOC8288834
LOC8260870	2.770975311	1.08E-08	4.43E-06	-
LOC107262116	3.17062083	8.67E-05	0.007128032	auxin-induced protein 15A-like
LOC8273022	1.648877568	0.00079935	0.03601133	putative glycine-rich cell wall structural protein 1
LOC8271822	2.07383798	0.000403625	0.0226671	uncharacterized LOC8271822

LOC8268530	3.284477338	2.40E-08	8.50E-06	uncharacterized LOC8268530
LOC8278416	1.654919757	0.00092652	0.040230679	putative ankyrin repeat protein RF_0381
LOC8267717	2.992443944	0.000192743	0.013024814	3-hydroxyisobutyryl-CoA hydrolase-like protein 1%2C mitochondrial%2C transcript variant X2
LOC8287674	1.467576365	0.000212246	0.014017844	ABC transporter G family member 15
LOC8285076	2.411515379	0.000402054	0.022666059	cyclic nucleotide-gated ion channel 2
LOC8265506	1.676299466	0.000542663	0.02775607	GATA zinc finger domain-containing protein 21
LOC8275528	1.218813199	0.0004217	0.023072532	RING-H2 finger protein ATL65
LOC107262123	1.292599016	0.000610518	0.030022775	uncharacterized LOC107262123
LOC8287551	6.299265776	1.77E-09	9.26E-07	uncharacterized LOC8287551
LOC8258445	1.366291269	0.000417938	0.022971212	squamosa promoter-binding-like protein 3
LOC8272658	2.821523379	0.000185198	0.012598427	beta-glucosidase 12
LOC8267260	4.172693343	6.42E-07	0.000137904	salicylic acid-binding protein 2
LOC107260799	4.895670598	5.82E-05	0.005275673	auxin-induced protein 15A-like
LOC8263577	5.137454578	1.36E-08	5.13E-06	GDSL esterase/lipase 2
LOC8284042	4.683674892	5.18E-08	1.58E-05	protein SRG1
LOC8278843	3.524016742	3.00E-09	1.39E-06	transcription factor SPATULA
LOC8279140	2.907664859	3.11E-06	0.000524998	phosphatidylinositol/phosphatidylcholine transfer protein SFH12%2C transcript variant X1
LOC8259069	4.868342658	3.41E-05	0.003388776	ethylene-responsive transcription factor ERF017
LOC8266005	3.943788873	3.59E-08	1.16E-05	subtilisin-like protease SBT1.3
LOC8258758	2.06976809	1.87E-05	0.002246241	uncharacterized LOC8258758
LOC8271128	4.825171776	4.32E-06	0.000653391	secoisolariciresinol dehydrogenase
LOC8275394	3.070543647	1.97E-05	0.00229683	calcium-binding protein KRP1
LOC8279485	2.129097311	0.001082624	0.045090175	tetraspanin-19%2C transcript variant X1
LOC8274243	2.56373929	0.000426751	0.023200223	GDSL esterase/lipase At2g30310-like
LOC8289351	2.668111441	0.000782625	0.035651369	trihelix transcription factor GTL2
LOC8273786	2.57153072	0.000689944	0.032821377	ABC transporter G family member 36-like
LOC8276602	1.261333283	0.000229435	0.014859211	L-type lectin-domain containing receptor kinase S.4

LOC8274189	2.455394675	0.000903355	0.039592785	formin-like protein 18%2C transcript variant X1
LOC8260346	1.361391586	0.000124806	0.009356628	protein SIEVE ELEMENT OCCLUSION B
LOC8283974	2.618175244	9.47E-08	2.54E-05	alpha carbonic anhydrase 7
LOC8286708	2.690309454	0.000142011	0.010350554	BAG family molecular chaperone regulator 6
LOC107261493	2.015144758	1.61E-06	0.000290002	uncharacterized LOC107261493
LOC8260681	3.062297612	7.00E-07	0.000145716	flavonol sulfotransferase-like
LOC107261577	4.484499159	0.00011036	0.008563628	auxin-responsive protein SAUR21-like
LOC8286786	1.632164166	0.00056733	0.028467104	4-coumarate--CoA ligase-like 1%2C transcript variant X1
LOC8261125	1.572145889	0.000807479	0.036251874	FT-interacting protein 1
LOC8266065	2.331139239	0.000228053	0.014822019	acid beta-fructofuranosidase 1%2C vacuolar
LOC8273667	2.093206184	4.13E-06	0.000633766	cinnamoyl-CoA reductase 1
LOC8261488	1.303522206	0.000166112	0.011659156	uncharacterized LOC8261488
LOC8273843	2.56715674	0.000550145	0.027994046	serine carboxypeptidase-like 7
LOC8258323	4.206272013	1.51E-06	0.000277342	uncharacterized LOC8258323%2C transcript variant X1
LOC8280868	3.191053492	1.30E-06	0.000246373	-
LOC8281712	3.418618029	5.60E-08	1.66E-05	probable 2-oxoglutarate/Fe(II)-dependent dioxygenase
LOC8287077	5.375463592	3.34E-05	0.003359521	formin-like protein 11
LOC8278542	4.233834493	9.20E-06	0.001234661	uncharacterized protein At4g04980
LOC8267219	3.605194923	1.69E-14	3.14E-11	CBL-interacting serine/threonine-protein kinase 20
LOC8265365	5.954662092	1.88E-07	4.63E-05	protein ESKIMO 1
LOC8279973	2.419121663	0.000127608	0.009435587	exocyst complex component EXO70H1
LOC8277307	2.823110451	9.66E-06	0.00127243	cytochrome P450 85A
LOC8268931	1.328681613	0.001014665	0.043140191	uncharacterized LOC8268931
LOC8274965	2.450311404	1.53E-05	0.001913935	uncharacterized LOC8274965
LOC8258651	5.959840406	6.70E-07	0.000141829	leucine-rich repeat receptor-like protein kinase PXL1
LOC8266551	4.488448875	9.10E-06	0.001234661	cinnamoyl-CoA reductase 1
LOC8282159	9.167832502	3.64E-11	3.23E-08	subtilisin-like protease SBT3.18
LOC8280432	7.453787174	2.46E-09	1.20E-06	legumin B
LOC107261253	4.508998114	1.58E-05	0.001956901	glycine-rich cell wall structural protein-like

LOC8289793	2.075822796	1.19E-06	0.00023078	carbonic anhydrase 2
LOC8269867	1.71597115	0.000104113	0.008203591	histone chaperone ASF1
LOC8268831	2.136730809	5.87E-05	0.005300541	uncharacterized LOC8268831
LOC8285824	2.328314086	2.94E-07	6.73E-05	uncharacterized LOC8285824
LOC8261722	3.173662255	2.09E-05	0.002379545	probable cysteine protease RD19D
LOC8285851	4.646499234	8.01E-05	0.006735005	laccase-21
LOC8286846	4.055567872	1.14E-08	4.56E-06	meiotic recombination protein SPO11-1
LOC8276095	1.781186482	0.000431132	0.023200223	alpha/beta hydrolase domain-containing protein 17B
LOC8269384	3.21611572	0.000106137	0.008299037	short-chain dehydrogenase TIC 32%2C chloroplastic
LOC8281161	1.594103431	0.000414062	0.022971212	cation/calcium exchanger 4
LOC8273845	3.984583235	0.000157311	0.011225194	serine carboxypeptidase-like 2
LOC8266261	2.456922238	0.000907258	0.039647355	protein CNGC15c
LOC8266891	2.553532367	2.62E-05	0.002833245	-
LOC8282525	3.086491097	0.000256409	0.016200601	class V chitinase
LOC8271862	6.347584364	2.85E-08	9.54E-06	ricin-like
LOC107261075	4.343178336	7.81E-06	0.001099114	receptor-like cytosolic serine/threonine-protein kinase RBK2%2C transcript variant X3
LOC8275549	2.44363957	0.000152697	0.01101142	cytosolic sulfotransferase 16
LOC8269193	7.841354667	5.36E-08	1.61E-05	11-beta-hydroxysteroid dehydrogenase-like 4A
LOC8265859	8.787491874	1.34E-08	5.13E-06	uncharacterized LOC8265859
LOC107261344	2.257155429	2.53E-05	0.002794745	floral homeotic protein AGAMOUS
LOC8284709	4.125777787	1.04E-08	4.34E-06	transcription factor MYB26
LOC8258612	4.903003414	0.000363391	0.020949384	ethylene-responsive transcription factor ERF017
LOC8258195	1.57640144	0.000203822	0.013590094	uncharacterized LOC8258195
LOC112536088	7.726795345	6.42E-06	0.000923026	probable 3-hydroxyisobutyryl-CoA hydrolase 2
LOC8281186	5.127319879	0.000137927	0.010088926	auxin-binding protein ABP19a
LOC107262117	5.028305424	0.000771635	0.035308363	auxin-induced protein 15A-like
LOC8283995	1.659942791	0.001171692	0.047538554	uncharacterized LOC8283995

LOC8287628	5.428455215	5.22E-06	0.00076135	endoglucanase 11%2C transcript variant X2
LOC8258322	3.381545275	4.63E-05	0.004393651	MADS-box protein AGL42
LOC8260347	1.623308064	0.000671853	0.032261591	uncharacterized LOC8260347
LOC107262006	2.403856804	0.000181392	0.012380743	uncharacterized LOC107262006
LOC8272405	2.069444092	0.000570514	0.028467104	-
LOC8281428	7.53948844	1.70E-06	0.000305129	laccase-14-like
LOC8267392	2.529410053	7.62E-07	0.000157132	cysteine-rich receptor-like protein kinase 15%2C transcript variant X1
LOC8266878	8.45911267	1.89E-09	9.43E-07	auxin-responsive protein SAUR68
LOC8287620	3.1762528	2.75E-05	0.002942131	uncharacterized LOC8287620
LOC8266881	8.432595242	8.27E-11	6.49E-08	auxin-responsive protein SAUR68
LOC8263324	2.574746139	2.81E-05	0.00299083	secoisolariciresinol dehydrogenase
LOC8285710	2.955730714	0.000166249	0.011659156	indole-3-acetic acid-amido synthetase GH3.17
LOC8269704	3.343357468	0.000211217	0.013995155	aluminum-activated malate transporter 9
LOC107261586	8.247422218	2.06E-08	7.64E-06	auxin-responsive protein SAUR21-like
LOC8285105	2.503711555	0.001233906	0.049278952	endoglucanase 9
LOC8277929	3.091411778	2.41E-06	0.000412922	glutamate receptor 2.7
LOC8279088	2.404732198	0.00013459	0.009915951	fatty-acid-binding protein 1
LOC8289459	2.457109674	0.0005667	0.028467104	transcription factor SPATULA%2C transcript variant X1
LOC107262111	3.754050266	0.000195146	0.013100455	auxin-induced protein 15A-like (Aux15A)
LOC8273228	2.841148616	0.000181381	0.012380743	serine carboxypeptidase-like 18
LOC107260723	6.517431521	1.10E-05	0.001430232	pectinesterase
LOC8262990	2.115314932	3.02E-05	0.003130137	uncharacterized LOC8262990
LOC8258234	2.482352846	0.000642644	0.031078386	calcium-dependent protein kinase 17
LOC8286127	2.706896112	0.000414117	0.022971212	protein DETOXIFICATION 41
LOC8258717	1.840632302	0.000712528	0.033351543	glucan endo-1%2C3-beta-glucosidase
LOC8268036	3.318039519	0.000843427	0.037418828	7-deoxyloganetin glucosyltransferase
LOC8266894	8.010340356	3.85E-09	1.71E-06	auxin-responsive protein SAUR68

LOC8267967	2.209062569	0.00116807	0.047486011	G-type lectin S-receptor-like serine/threonine-protein kinase LECRK3
LOC8280449	2.766329169	2.58E-05	0.002833245	potassium channel SKOR
LOC8266295	3.480462211	0.001102224	0.045627152	transcription factor MYC2
LOC8268038	4.628709968	0.000362123	0.020935439	7-deoxyloganetin glucosyltransferase
LOC107261581	7.976123206	2.32E-07	5.44E-05	auxin-responsive protein SAUR21-like
LOC8266888	2.56214257	0.000304958	0.018275028	auxin-responsive protein SAUR68
LOC107261587	7.935951256	3.59E-06	0.000576842	auxin-responsive protein SAUR21-like
LOC8285300	4.776558553	1.64E-05	0.002014417	double-stranded RNA-binding protein 3%2C transcript variant X2
LOC8274777	7.897580343	3.32E-08	1.09E-05	transcription factor PAR2
LOC8283214	7.806212315	3.45E-06	0.000558606	auxin-responsive protein SAUR63-like
LOC107261578	6.830684962	4.57E-06	0.000686499	auxin-responsive protein SAUR21-like
LOC8273267	6.81926632	4.69E-07	0.00010404	agglutinin-like
LOC8283211	3.696728429	0.000262525	0.016334163	auxin-responsive protein SAUR62
LOC8260126	5.787200582	1.52E-05	0.001909985	vinorine synthase
LOC8270745	7.687225243	0.000108754	0.008471169	uncharacterized LOC8270745
LOC8274301	3.909379979	0.00068889	0.032821377	-
LOC8267061	7.653875689	7.20E-08	2.04E-05	uncharacterized LOC8267061
LOC8283212	6.678197696	2.13E-06	0.000367528	auxin-responsive protein SAUR63
LOC8266431	3.210424646	5.20E-05	0.004843457	anti-sigma-I factor RsgI6
LOC8276902	7.558186435	1.61E-07	4.00E-05	zinc-finger homeodomain protein 3
LOC8287285	5.629676803	1.10E-05	0.001430232	oligopeptide transporter 1
LOC8282905	3.384965884	0.001025255	0.043230183	-
LOC8266882	6.531012521	1.21E-06	0.000233864	auxin-responsive protein SAUR68
LOC8260311	4.304723481	1.94E-05	0.00229683	phospholipase A1-Igamma1%2C chloroplastic
LOC8289049	3.340539482	6.87E-05	0.006019527	transcription factor TCP13
LOC8263434	3.811069047	8.61E-05	0.007116483	gibberellin-regulated protein 6
LOC8276874	4.812471909	7.45E-05	0.006442727	monoterpene synthase

LOC8268009	4.313421772	9.28E-05	0.007455506	protein GAST1
LOC8269097	6.384964087	2.24E-05	0.002527492	serine carboxypeptidase-like 40
LOC8285885	4.105570772	0.000125809	0.009356628	glycine-rich cell wall structural protein 1
LOC8273054	4.560515176	1.87E-05	0.002246241	heavy metal-associated isoprenylated plant protein 35
LOC8266895	7.277383977	3.76E-07	8.44E-05	auxin-responsive protein SAUR68
LOC8271759	4.345517542	8.70E-05	0.007128032	nuclear transcription factor Y subunit B-4
LOC8265439	7.205557267	1.54E-05	0.001913935	lignin-forming anionic peroxidase
LOC8276939	7.126534015	4.75E-07	0.000104282	uncharacterized LOC8276939
LOC8287432	2.484203754	0.001064442	0.044606029	heavy metal-associated isoprenylated plant protein 39
LOC8274392	3.160890254	0.000338311	0.019896998	linoleate 13S-lipoxygenase 2-1%2C chloroplastic
LOC8266875	6.060357342	3.17E-05	0.003219665	auxin-responsive protein SAUR68
LOC8280176	2.999866878	0.00098868	0.042331628	E3 ubiquitin protein ligase DRIP2
LOC8287215	4.723549862	0.000383616	0.021868239	transcription repressor OFP16
LOC8287938	4.590612908	8.92E-05	0.007283077	serine carboxypeptidase-like 18
LOC8283240	5.199109106	7.93E-05	0.00671649	uncharacterized LOC8283240
LOC8277590	6.892273906	1.17E-05	0.001505492	fatty acyl-CoA reductase 3
LOC8266880	4.945870096	0.000431992	0.023200223	auxin-responsive protein SAUR68
LOC112533688	3.641520295	0.001204876	0.048595087	uncharacterized LOC112533688
LOC8281589	6.764590781	0.000423262	0.023096068	probable LRR receptor-like serine/threonine-protein kinase At1g51860%2C transcript variant X3
LOC8266887	6.710320818	4.03E-06	0.00062741	auxin-responsive protein SAUR68
LOC8258525	4.930996083	0.00070059	0.032976057	BON1-associated protein 2%2C transcript variant X1
LOC8269871	4.375319526	0.000629953	0.030609735	polygalacturonase At1g48100
LOC8258576	6.434580876	0.000568631	0.028467104	trihelix transcription factor GTL1
LOC8283213	5.393343789	0.000344885	0.020109753	auxin-responsive protein SAUR68
LOC8271129	6.324525678	0.000499933	0.026160581	tropinone reductase-like 1
LOC8278136	3.864437241	0.000792707	0.035791074	zinc finger protein 2
LOC107262113	6.189082994	0.000194253	0.013083545	auxin-induced protein 15A-like
LOC8266876	6.118153193	0.000258643	0.016283135	auxin-responsive protein SAUR68

LOC8283270	6.064979994	0.000458058	0.024407453	la-related protein 6C%2C transcript variant X1
LOC8262767	5.999593557	0.000612683	0.030056802	acanthoscurrin-2
LOC8269101	5.98179079	0.000180064	0.012372857	-
LOC8281177	5.877594921	0.000418723	0.022971212	uncharacterized LOC8281177%2C transcript variant X1
LOC8265202	5.729107452	0.000569041	0.028467104	-
LOC8266884	5.705820736	0.000788599	0.035763859	auxin-responsive protein SAUR68
LOC8281992	5.550284225	0.001085874	0.045133445	wall-associated receptor kinase 5
