# Supplementary material for: Lupeol Accumulation Correlates with Auxin in the Epidermis of Castor
Source: Molecules. 2021 May 17;26(10):2978. doi: 10.3390/molecules26102978 (PMC8156332; doi:10.3390/molecules26102978)
Supplement: Supplementary file 1 [file molecules-26-02978-s001.zip › Supplemental figures and Tables/Table S4.pdf]

Table S4. Downregulated genes in the first internode of stem compared to upper hypocotyl of 337 individual.

Gene-ID	log2FoldChange	p-value	padj	Gene description
LOC107262069	-4.592540885	6.22E-15	1.41E-11	uncharacterized LOC107262069
LOC8265844	-6.50524401	2.62E-14	4.45E-11	putative expansin-B2
LOC8269032	-3.488913543	7.45E-14	1.17E-10	cationic amino acid transporter 6%2C chloroplastic
LOC8286250	-4.162987908	3.23E-13	4.11E-10	cellulose synthase-like protein G3
LOC8265434	-5.23276661	6.41E-12	6.54E-09	lignin-forming anionic peroxidase
LOC8268766	-3.673736195	1.24E-11	1.21E-08	dirigent protein 19
LOC8258439	-2.617872311	1.13E-10	8.56E-08	ethylene-responsive transcription factor 12
LOC8262900	-1.900224928	2.85E-10	1.76E-07	uncharacterized LOC8262900
LOC112536839	-2.345642732	1.33E-09	7.36E-07	root meristem growth factor 6-like
LOC8274141	-2.40371232	1.39E-09	7.49E-07	probable calcium-binding protein CML29
LOC8273735	-2.384175054	6.49E-09	2.76E-06	cytochrome P450 94C1
LOC112534258	-4.449600302	2.60E-08	8.98E-06	uncharacterized LOC112534258
LOC112536574	-3.31092266	8.21E-08	2.26E-05	uncharacterized LOC112536574
LOC8273209	-5.355541872	9.92E-08	2.63E-05	probable purine permease 4
LOC107261747	-2.086990261	1.20E-07	3.10E-05	uncharacterized LOC107261747%2C transcript variant X2
LOC8258914	-2.693524349	1.51E-07	3.81E-05	protein DETOXIFICATION 54
LOC8272130	-2.045947369	9.13E-07	0.00018275	geraniol 8-hydroxylase
LOC8284056	-1.942345992	1.15E-06	0.00022703	purine permease 21
LOC8259697	-2.279619403	1.16E-06	0.00022703	transcription factor IBH1
LOC8277511	-3.838636353	1.27E-06	0.00024275	3%2C9-dihydroxypterocarpan 6A-monooxygenase
LOC8276207	-2.829231148	1.38E-06	0.00025929	uncharacterized LOC8276207
LOC8270419	-3.010627556	1.86E-06	0.00032954	basic blue protein
LOC8285096	-2.262731688	1.87E-06	0.00032954	uncharacterized LOC8285096
LOC107261255	-3.691358387	2.06E-06	0.00035877	uncharacterized LOC107261255
LOC8259917	-2.276749897	2.69E-06	0.00045833	protein EXORDIUM-like 2
LOC112536123	-2.048748329	3.16E-06	0.00052916	uncharacterized LOC112536123
LOC8258460	-6.94047647	3.43E-06	0.00055861	transcription factor bHLH162

LOC8258458	-1.608919802	3.68E-06	0.00058715	non-classical arabinogalactan protein 31
LOC8273472	-2.706286941	3.89E-06	0.00061522	probable anion transporter 3%2C chloroplastic
LOC8265495	-1.687432441	3.94E-06	0.00061799	berberine bridge enzyme-like 13
LOC8277524	-2.482275938	4.67E-06	0.00069509	putative GATA transcription factor 22
LOC8258887	-2.933621199	4.83E-06	0.00071357	probable purine permease 4
LOC8281676	-2.414087695	4.94E-06	0.00072585	phospho-2-dehydro-3-deoxyheptonate aldolase 1%2C chloroplastic
LOC8278139	-3.037838532	7.01E-06	0.00100011	glucan 1%2C3-beta-glucosidase A-like
LOC8284503	-3.307257056	8.00E-06	0.00111886	transcription factor TCP11
LOC8275462	-2.384727398	8.13E-06	0.00112841	germin-like protein subfamily 3 member 4
LOC8272477	-2.330132545	9.21E-06	0.00123466	subtilisin-like protease SBT1.7
LOC8267806	-2.384746086	9.63E-06	0.00127243	glucomannan 4-beta-mannosyltransferase 9
LOC8275089	-2.469599211	1.36E-05	0.0017439	lipid phosphate phosphatase 1
LOC8280934	-1.927129519	1.37E-05	0.00175195	uncharacterized LOC8280934
LOC8275787	-2.978013419	1.69E-05	0.00206585	cytokinin dehydrogenase 5
LOC8282965	-2.566927657	1.98E-05	0.00229683	caffeic acid 3-O-methyltransferase 1
LOC8283996	-1.549975177	2.03E-05	0.0023377	S-adenosylmethionine synthase 3%2C transcript variant X1
LOC8267024	-2.359060571	2.06E-05	0.0023622	arogenate dehydratase/prephenate dehydratase 6%2C chloroplastic
LOC8264854	-3.145546468	2.19E-05	0.00247939	alpha carbonic anhydrase 7
LOC8284759	-1.164881834	2.34E-05	0.00262699	uncharacterized LOC8284759
LOC112535464	-2.943052349	2.46E-05	0.00273329	small nucleolar RNA U3
LOC112534372	-2.692285789	2.61E-05	0.00283325	uncharacterized LOC112534372
LOC8258697	-3.200087275	2.62E-05	0.00283325	protein SRG1
TRNAV-AAC	-2.500672381	2.64E-05	0.00283661	tRNA-Val
LOC8275968	-2.404091259	2.85E-05	0.00301722	uncharacterized LOC8275968
LOC8289448	-2.223118714	2.97E-05	0.00310526	uncharacterized LOC8289448
LOC8269507	-2.322913833	3.05E-05	0.0031438	CLAVATA3/ESR (CLE)-related protein 12
LOC8263958	-4.916304454	3.10E-05	0.0031722	uncharacterized LOC8263958

LOC8271908	-1.823239946	3.11E-05	0.0031722	transcription factor TGA9
LOC8281705	-2.682145376	3.24E-05	0.0032713	blue copper protein
LOC8272127	-2.685475007	3.41E-05	0.00338878	protein ODORANT1
LOC107261168	-1.73734551	3.42E-05	0.00338878	auxin-responsive protein SAUR50-like
LOC8284724	-2.995254693	3.92E-05	0.00384919	cytochrome P450 84A1-like
LOC8281597	-2.175645503	3.97E-05	0.00388075	inositol oxygenase 1
LOC8289134	-1.477399399	4.04E-05	0.00392895	probable WRKY transcription factor 57
LOC8286038	-1.61710844	4.51E-05	0.00432319	tetraspanin-3
LOC8282005	-2.246283686	4.60E-05	0.00439017	trans-cinnamate 4-monooxygenase
LOC8272420	-2.079188953	4.84E-05	0.00455181	protein IQ-DOMAIN 1
LOC8278901	-1.729221169	5.32E-05	0.00491149	uncharacterized LOC8278901
LOC8270475	-1.980704458	6.35E-05	0.00565935	chlorophyll a-b binding protein CP29.3%2C chloroplastic
LOC8272446	-1.365400267	6.56E-05	0.00582135	AT-hook motif nuclear-localized protein 1
LOC8270892	-1.258371475	6.68E-05	0.00587676	uncharacterized protein At5g39865
LOC8285447	-1.878552514	6.93E-05	0.00602302	uncharacterized LOC8285447
LOC107262171	-2.932593395	6.94E-05	0.00602302	FGGY carbohydrate kinase domain-containing protein-like%2C transcript variant X1
LOC8269157	-3.128008911	7.50E-05	0.0064566	leucine-rich repeat extensin-like protein 4
LOC8261648	-1.668962197	7.69E-05	0.00656687	inactive TPR repeat-containing thioredoxin TTL3
LOC8274851	-2.990846935	7.80E-05	0.00663568	vascular-related unknown protein 1
LOC8259521	-2.496480601	8.02E-05	0.00673501	LOB domain-containing protein 15
LOC8279575	-2.623728249	8.23E-05	0.00688708	RING-H2 finger protein ATL51
LOC8284504	-1.07518292	8.33E-05	0.00693752	RNA polymerase sigma factor sigF%2C chloroplastic
LOC8268240	-2.831020613	8.54E-05	0.00708865	uncharacterized LOC8268240%2C transcript variant X3
LOC8280867	-2.485928019	8.98E-05	0.00728803	leucine-rich repeat extensin-like protein 6
LOC8263079	-1.683721713	9.00E-05	0.00728803	uncharacterized LOC8263079
LOC8289723	-1.281597258	9.20E-05	0.00741939	protein DETOXIFICATION 12
LOC8271389	-1.374018469	9.40E-05	0.00752584	acyl-protein thioesterase 2
LOC8279653	-1.467039212	9.75E-05	0.00774615	lipid transfer protein EARLI 1

LOC8261800	-2.299711358	9.89E-05	0.0078229	uncharacterized LOC8261800
LOC8261912	-2.328697781	0.00011399	0.00872868	delta-like protein 4
LOC8274952	-1.878992015	0.0001141	0.00872868	S-adenosylmethionine synthase 1
LOC8258462	-1.734491215	0.0001142	0.00872868	2-methylene-furan-3-one reductase%2C transcript variant X2
LOC8266063	-1.760230572	0.00011756	0.00891863	NAC domain-containing protein 7
LOC8280259	-2.092366272	0.00012608	0.00935663	shikimate O-hydroxycinnamoyltransferase
LOC8270244	-1.745087331	0.00014692	0.01066999	nodulation receptor kinase
LOC8285306	-2.294956264	0.00015334	0.01101901	DELLA protein RGL1
LOC8275623	-1.51465093	0.00016717	0.01168365	probable protein phosphatase 2C 72
LOC112536661	-3.888053284	0.00017192	0.01189319	uncharacterized LOC112536661
LOC8267676	-1.266903599	0.00017394	0.01199262	inactive LRR receptor-like serine/threonine-protein kinase BIR2
LOC8286431	-3.691231109	0.00018608	0.01261606	60S acidic ribosomal protein P0
LOC8282762	-1.985183803	0.00020269	0.01356199	spermidine coumaroyl-CoA acyltransferase
LOC8275067	-2.131249331	0.00021673	0.01426755	uncharacterized LOC8275067%2C transcript variant X1
LOC8268805	-1.565233524	0.00022148	0.01445302	tubulin beta-1 chain-like
LOC8286491	-3.679845635	0.00023008	0.01485921	protein EMSY-LIKE 4%2C transcript variant X3
LOC8259529	-1.650122873	0.00023263	0.01492899	adenine/guanine permease AZG1
LOC8272096	-1.589299682	0.00023677	0.01514713	abscisic acid receptor PYL4
LOC107261969	-1.471775999	0.00024713	0.01576063	fatty acid amide hydrolase-like
LOC8280572	-3.054610135	0.00025162	0.01599699	protein NRT1/ PTR FAMILY 2.11%2C transcript variant X1
LOC8258787	-2.085288179	0.0002527	0.01601555	chitinase-like protein 2
LOC8280708	-2.333263947	0.00025931	0.01628314	probable galacturonosyltransferase 12
LOC8269693	-2.602462908	0.00026041	0.01630219	uncharacterized LOC8269693
LOC8264956	-1.552544278	0.00027728	0.01716796	cytochrome b5
LOC8260939	-2.242045081	0.00027761	0.01716796	pathogenesis-related protein 5
LOC8280301	-1.657688427	0.00028255	0.01742068	4-coumarate--CoA ligase 2
LOC8271157	-1.586539704	0.00029302	0.01794965	protein JASON%2C transcript variant X1

LOC8268088	-2.62854931	0.00029377	0.01794965	E3 ubiquitin-protein ligase RHA2A
LOC8264689	-2.085865746	0.00030234	0.01827503	flavonol sulfotransferase-like
LOC8260907	-3.646342281	0.00030514	0.01827503	cytochrome P450 CYP82D47
LOC107261485	-3.87642635	0.00030536	0.01827503	S-protein homolog 74-like
LOC8272614	-1.574208816	0.00031803	0.01892205	LOB domain-containing protein 4
LOC8266050	-1.984242767	0.00032076	0.01902945	protein ODORANT1
LOC8278631	-1.834638063	0.00032537	0.01924708	protein trichome birefringence-like 41%2C transcript variant X2
LOC8269391	-2.455116556	0.00032827	0.01936199	probable aquaporin PIP1-2
LOC8287174	-1.532637875	0.00034313	0.02009298	monothiol glutaredoxin-S9-like
LOC112537093	-3.126047986	0.00037986	0.0217149	uncharacterized LOC112537093
LOC8285003	-2.099334347	0.00039768	0.02260665	extradiol ring-cleavage dioxygenase
LOC8266762	-1.221913277	0.00040169	0.02266606	cytochrome b561 and DOMON domain-containing protein At3g25290
LOC8275407	-1.91344133	0.00041009	0.02292881	VAN3-binding protein%2C transcript variant X2
LOC8267683	-1.721722474	0.00041688	0.02297121	uncharacterized LOC8267683
LOC8288683	-1.585768985	0.00041701	0.02297121	uncharacterized LOC8288683
LOC8285670	-1.612594791	0.00041849	0.02297121	protein LAX PANICLE 2%2C transcript variant X1
LOC8288060	-2.636909608	0.00047647	0.02525674	geraniol 8-hydroxylase-like
LOC8273543	-1.824296181	0.00047903	0.02532649	phenylalanine ammonia-lyase G4
LOC8284203	-1.776795781	0.00049264	0.02597886	uncharacterized LOC8284203
LOC8277880	-1.57325414	0.00049891	0.02616058	cytochrome P450 98A2
LOC8284829	-1.380550865	0.00049976	0.02616058	uncharacterized LOC8284829
LOC8288434	-6.149869917	0.00050731	0.02634408	cysteine synthase%2C transcript variant X1
LOC8262224	-1.41832204	0.00051044	0.0264394	alcohol dehydrogenase-like 6%2C transcript variant X1
LOC8285642	-1.57795138	0.00051342	0.02652626	probable beta-1%2C4-xylosyltransferase IRX14
LOC8278456	-1.316908991	0.00051714	0.02665076	TLC domain-containing protein At5g14285
LOC8267082	-1.468809147	0.0005263	0.0270548	uncharacterized LOC8267082%2C transcript variant X1
LOC112535736	-2.805266684	0.0005405	0.02771477	uncharacterized LOC112535736%2C transcript variant X2

LOC107261150	-2.918474692	0.00054904	0.02799405	uncharacterized LOC107261150
LOC8260554	-1.099604826	0.00056501	0.0284671	dof zinc finger protein DOF3.4
LOC8288340	-1.534166999	0.00056937	0.0284671	caffeoyl-CoA O-methyltransferase
LOC8261059	-2.198761131	0.00057537	0.02863923	cytochrome P450 CYP73A100
LOC8288079	-1.723418887	0.00057793	0.02869704	tubulin alpha chain
LOC107261706	-1.779615604	0.00058001	0.02872998	non-specific lipid-transfer protein 2
LOC8272600	-2.27537045	0.00060406	0.029849	auxin-responsive protein SAUR71
LOC8285147	-2.592385672	0.00067014	0.0322552	-
LOC8287082	-1.798708756	0.00068536	0.03282138	rapid alkalinization factor
LOC8259090	-2.999467148	0.00068827	0.03282138	F-box/kelch-repeat protein At3g23880
LOC8277848	-2.040926528	0.00069307	0.03289141	alcohol dehydrogenase
LOC8266484	-1.948746209	0.00070178	0.03297606	cellulose synthase A catalytic subunit 4 [UDP-forming]%2C transcript variant X3
LOC8272135	-2.225474353	0.00073544	0.03434505	primary amine oxidase
LOC8267792	-1.473800782	0.00073954	0.03443903	dirigent protein 19
LOC8285813	-1.792484545	0.00074082	0.03443903	uncharacterized LOC8285813
LOC8265886	-1.317403041	0.00074532	0.03455923	protein RADIALIS-like 3
LOC8263188	-1.474274236	0.0007468	0.03455923	rho GTPase-activating protein 5
LOC8261561	-2.146213827	0.00075712	0.03487896	ATP synthase subunit alpha%2C mitochondrial
LOC8280189	-1.258063395	0.00076536	0.0350999	CBL-interacting serine/threonine-protein kinase 7
LOC8259579	-1.984886579	0.00077698	0.03547352	NDR1/HIN1-like protein 13
LOC8282179	-4.976700376	0.00083196	0.03699062	probable transcription repressor OFP9
LOC8274298	-1.953425362	0.00086529	0.03822263	CRIB domain-containing protein RIC4
LOC8269562	-1.803233065	0.00086758	0.03824108	uncharacterized LOC8269562
LOC8289722	-1.830236187	0.00090407	0.03959279	protein DETOXIFICATION 12%2C transcript variant X1
LOC107262651	-1.87306422	0.00091015	0.03968861	probable beta-1%2C4-xylosyltransferase IRX14
LOC112536202	-1.974363671	0.00091505	0.03981728	uncharacterized LOC112536202
LOC8267311	-2.222807496	0.00093394	0.040456	probable WRKY transcription factor 23
LOC8287424	-1.924551924	0.00096091	0.04137197	protein SPIRAL1-like 5

LOC8285899	-1.431402913	0.00098374	0.0422654	protein usf
LOC8282708	-1.720679929	0.0009915	0.04233163	silicon efflux transporter LSI2%2C transcript variant X2
LOC8261159	-1.595415537	0.00101987	0.04323018	O-acyltransferase WSD1
LOC8258330	-1.843043703	0.00102491	0.04323018	carboxylesterase 1
LOC8276167	-2.612258716	0.00103413	0.0435146	AT-hook motif nuclear-localized protein 20
LOC8258580	-1.287903915	0.00104552	0.04390312	cytokinin dehydrogenase 7
LOC8282880	-2.064656216	0.00112797	0.04641036	aspartic proteinase nepenthesin-1
LOC8272075	-2.024958809	0.00114006	0.04671958	sister chromatid cohesion 1 protein 1
LOC8285762	-1.8534219	0.00115355	0.0471777	cytochrome b561 and DOMON domain-containing protein At5g47530
LOC8283095	-6.021969865	0.0011635	0.04743372	putative UPF0481 protein At3g02645
LOC8259214	-1.372780463	0.00116446	0.04743372	phospholipase A1-IIgamma
LOC8258600	-1.210436664	0.00117443	0.04755516	monoacylglycerol lipase ABHD6
LOC8282879	-1.452800359	0.00123231	0.04927895	uncharacterized LOC8282879
