# Supplementary material for: Lupeol Accumulation Correlates with Auxin in the Epidermis of Castor
Source: Molecules. 2021 May 17;26(10):2978. doi: 10.3390/molecules26102978 (PMC8156332; doi:10.3390/molecules26102978)
Supplement: Supplementary file 1 [file molecules-26-02978-s001.zip › Supplemental figures and Tables/Table S5.pdf]

Table S5. GO categories significantly enriched in the first internode of stem of 337 individual. The results are grouped into the molecular function (MF) and biological process (BP).

Category	GOID	Description	GeneRatio	padj	Count	Up	Up_Gene_names	Down	Down_Gene_names
BP	GO:0009733	response to auxin	22/186	3.83E-17	22	20	8266881/8266878/8266894/107261586/107261581/8266895/8266882/8283212/8283214/8266887/107261578/8266875/107261577/107262111/8266876/8283211/8266888/8283213/8266880/8266884	2	107261168/8272600
BP	GO:0009719	response to endogenous stimulus	22/186	8.81E-15	22	20	8266881/8266878/8266894/107261586/107261581/8266895/8266882/8283212/8283214/8266887/107261578/8266875/107261577/107262111/8266876/8283211/8266888/8283213/8266880/8266884	2	107261168/8272600
BP	GO:0009725	response to hormone	22/186	8.81E-15	22	20	8266881/8266878/8266894/107261586/107261581/8266895/8266882/8283212/8283214/8266887/107261578/8266875/107261577/107262111/8266876/8283211/8266888/8283213/8266880/8266884	2	107261168/8272600

BP	GO:0010033	response to organic substance	22/186	8.81E-15	22	20	8266881/8266878/8266894/107261586/107261581/8266895/8266882/8283212/8283214/8266887/107261578/8266875/107261577/107262111/8266876/8283211/8266888/8283213/8266880/8266884	2	107261168/8272600
BP	GO:0042221	response to chemical	26/186	3.63E-13	26	21	8266881/8266878/8266894/107261586/107261581/8266895/8266882/8283212/8283214/8266887/107261578/8266875/107261577/107262111/8266876/8283211/8266888/8283213/8286127/8266880/8266884	5	8258914/107261168/8289723/8272600/8289722
MF	GO:0005506	iron ion binding	17/216	0.002338	17	6	8266677/8284230/8277307/8261035/8287395/8284294	11	8273735/8272130/8277511/8284724/8281597/8282005/8260907/8285003/8288060/8277880/8261059
MF	GO:0016798	hydrolase activity, acting on glycosyl bonds	16/216	0.018594	16	14	8273266/8289192/8271862/8273267/8287628/8273057/8266431/8287425/8272658/8266065/8268051/8269871/8272272/8285105	2	8278139/8258787

MF	GO:0016705	oxidoreductase activity, acting on paired donors	14/216	0.018594	14	5	8284230/8277307/8261035/8287395/8284294	9	8273735/8272130/8277511/8284724/8282005/8260907/8288060/8277880/8261059
MF	GO:0020037	heme binding	16/216	0.018594	16	6	8284230/8277307/8265439/8261035/8287395/8284294	10	8265434/8273735/8272130/8277511/8284724/8282005/8260907/8288060/8277880/8261059
MF	GO:0046906	tetrapyrrole binding	16/216	0.018594	16	6	8284230/8277307/8265439/8261035/8287395/8284294	10	8265434/8273735/8272130/8277511/8284724/8282005/8260907/8288060/8277880/8261059
MF	GO:0016799	hydrolase activity, hydrolyzing N-glycosyl compounds	4/216	0.018594	4	4	8273266/8271862/8273267/8268051	0	
MF	GO:0003860	3-hydroxyisobutyryl-CoA hydrolase activity	3/216	0.02195	3	3	8266552/112536088/8267717	0	

MF	GO:0016289	CoA hydrolase activity	3/216	0.02195	3	3	8266552/112536088/8267717	0
MF	GO:0030597	RNA glycosylase activity	3/216	0.023203	3	3	8273266/8271862/8273267	0
MF	GO:0030598	rRNA N-glycosylase activity	3/216	0.023203	3	3	8273266/8271862/8273267	0
MF	GO:0005216	ion channel activity	5/216	0.023203	5	5	8264121/8277929/8265217/8280449/8266261	0
MF	GO:0015267	channel activity	5/216	0.023203	5	5	8264121/8277929/8265217/8280449/8266261	0
MF	GO:0022803	passive transmembrane transporter activity	5/216	0.023203	5	5	8264121/8277929/8265217/8280449/8266261	0
MF	GO:0022838	substrate-specific channel activity	5/216	0.023203	5	5	8264121/8277929/8265217/8280449/8266261	0

MF	GO:0004185	serine-type carboxypeptidase activity	5/216	0.023203	5	5	8269097/8287938/8273845/8273228/8273843	0	
MF	GO:0016747	transferase activity, transferring	9/216	0.023203	9	6	8280192/8283500/8260126/8289602/107261016/8277118	3	8280259/8282762/8261159
MF	GO:0004180	carboxypeptidase activity	5/216	0.024071	5	5	8269097/8287938/8273845/8273228/8273843	0	
MF	GO:0008236	serine-type peptidase activity	9/216	0.024071	9	8	8282159/8266005/8269097/8287938/8273845/8273228/8273843/8276338	1	8272477
MF	GO:0017171	serine hydrolase activity	9/216	0.024071	9	8	8282159/8266005/8269097/8287938/8273845/8273228/8273843/8276338	1	8272477
MF	GO:0016790	thiolester hydrolase activity	3/216	0.024863	3	3	8266552/112536088/8267717	0	

MF	GO:0070008	serine-type exopeptidase activity	5/216	0.026565	5	5	8269097/8287938/8273845/8273228/8273843	0	
MF	GO:0008146	sulfotransferase activity	3/216	0.026565	3	2	8260681/8275549	1	8264689
MF	GO:0016782	transferase activity, transferring sulfur-containing groups	3/216	0.026565	3	2	8260681/8275549	1	8264689
MF	GO:0016746	transferase activity, transferring acyl groups	10/216	0.027212	10	7	8280192/8263008/8283500/8260126/8289602/107261016/8277118	3	8280259/8282762/8261159
MF	GO:0016835	carbon-oxygen lyase activity	5/216	0.040111	5	4	8289793/8267425/8276874/8280754	1	8267024
MF	GO:0008238	exopeptidase activity	5/216	0.045043	5	5	8269097/8287938/8273845/8273228/8273843	0	
