# Supplementary material for: Lupeol Accumulation Correlates with Auxin in the Epidermis of Castor
Source: Molecules. 2021 May 17;26(10):2978. doi: 10.3390/molecules26102978 (PMC8156332; doi:10.3390/molecules26102978)
Supplement: Supplementary file 1 [file molecules-26-02978-s001.zip › Supplemental figures and Tables/Table S6.pdf]

Table S6. Enriched KEGG biochemical pathways in the first internode of the stem of 337 individual.

KEGGID	Description	GeneRatio	padj	Count	Up	Up_Gene_names	Down	Down_Gene_names
rcu00940	Phenylpropanoid biosynthesis	15/88	4.81E-06	15	4	8289465/8265439/8272658/8286786	11	8265434/8265495/8282965/8284724/8282005/8280259/8280301/8273543/8277880/8288340/8261059
rcu00999	Secondary metabolites biosynthesis	6/88	1.25E-05	6	2	8289465/8286786	4	8282005/8280301/8288340/8261059
rcu04075	Plant hormone signal transduction	17/88	2.94E-05	17	13	107261581/107261578/107260799/107262116/107261577/8285710/107262113/107262111/8280179/8273523/8271341/107262117/8266295	4	8271908/107261168/8272096/8272600
rcu00945	Stilbenoid biosynthesis	6/88	3.16E-05	6	1	8289465	5	8282005/8280259/8277880/8288340/8261059
rcu00941	Flavonoid biosynthesis	7/88	5.29E-05	7	2	8274087/8289465	5	8282005/8280259/8277880/8288340/8261059
rcu00591	Linoleic acid metabolism	3/88	0.036665439	3	3	8284230/8274394/8274392	0	
rcu00130	Terpenoid-quinone biosynthesis	4/88	0.050692058	4	1	8286786	3	8282005/8280301/8261059

rcu00073	Cutin and wax biosynthesis	3/88	0.063514031	3	3	8266677/8277590/8287395	0	
rcu00910	Nitrogen metabolism	3/88	0.105045255	3	2	8283974/8289793	1	8264854
rcu00909	Triterpenoid biosynthesis	2/88	0.106732658	2	2	8280320/8280754	0	
rcu00905	Brassinosteroid biosynthesis	2/88	0.160982706	2	2	8277307/8261035	0	
rcu00908	Zeatin biosynthesis	2/88	0.217355978	2	0		2	8275787/8258580
rcu00410	beta-Alanine metabolism	3/88	0.29258867	3	2	8266552/8267717	1	8272135
rcu00592	alpha-Linolenic acid metabolism	3/88	0.335117267	3	2	8274394/8274392	1	8277848
rcu00360	Phenylalanine metabolism	2/88	0.457991639	2	0		2	8273543/8272135
rcu00062	Fatty acid elongation	2/88	0.457991639	2	2	8280192/8283500	0	
rcu00350	Tyrosine metabolism	2/88	0.479970539	2	0		2	8277848/8272135
rcu04016	MAPK signaling pathway - plant	4/88	0.522968696	4	3	8273523/8258591/8266295	1	8272096
rcu00640	Propanoate metabolism	2/88	0.522968696	2	2	8266552/8267717	0	
rcu00561	Glycerolipid metabolism	3/88	0.522968696	3	2	8263008/8259873	1	8275089
rcu00400	Phenylalanine, tyrosine and tryptophan biosynthesis	2/88	0.624603619	2	0		2	8281676/8267024
rcu00564	Glycerophospholipid metabolism	3/88	0.624603619	3	1	8263008	2	8275089/8271389
rcu00280	Valine, leucine and isoleucine degradation	2/88	0.624603619	2	2	8266552/8267717	0	
rcu00500	Starch and sucrose metabolism	4/88	0.624603619	4	4	8272658/8266065/8288961/8285105	0	

rcu00196	Photosynthesis - antenna proteins	1/88	0.624603619	1	0		1	8270475
rcu00950	Isoquinoline alkaloid biosynthesis	1/88	0.624603619	1	0		1	8272135
rcu00270	Cysteine and methionine metabolism	3/88	0.651212822	3	0		3	8283996/8274952/8288434
rcu00520	Amino sugar and nucleotide sugar metabolism	3/88	0.710558636	3	3	8282525/8288961/8272272	0	
rcu00960	Tropane, piperidine and pyridine alkaloid biosynthesis	1/88	0.722937739	1	0		1	8272135
rcu01230	Biosynthesis of amino acids	5/88	0.813589554	5	0		5	8281676/8283996/8267024/8274952/8288434
rcu00920	Sulfur metabolism	1/88	0.819531064	1	0		1	8288434
rcu03020	RNA polymerase	1/88	0.819531064	1	0		1	112536123
rcu04626	Plant-pathogen interaction	3/88	0.819531064	3	3	8285076/8258234/8266261	0	
rcu00380	Tryptophan metabolism	1/88	0.819531064	1	1	8284230	0	
rcu00906	Carotenoid biosynthesis	1/88	0.819531064	1	1	8288174	0	
rcu03410	Base excision repair	1/88	0.819531064	1	1	8268051	0	
rcu00460	Cyanoamino acid metabolism	1/88	0.819531064	1	1	8272658	0	
rcu00053	Ascorbate and aldarate metabolism	1/88	0.819531064	1	0		1	8281597
rcu00020	Citrate cycle (TCA cycle)	1/88	0.819531064	1	1	8288718	0	
rcu00071	Fatty acid degradation	1/88	0.819531064	1	0		1	8277848
rcu00190	Oxidative phosphorylation	2/88	0.831363593	2	1	8284533	1	8261561
rcu00052	Galactose metabolism	1/88	0.831363593	1	1	8266065	0	

rcu00900	Terpenoid backbone biosynthesis	1/88	0.831363593	1	1	8258747	0	
rcu00260	Glycine, serine and threonine metabolism	1/88	0.831363593	1	0		1	8272135
rcu00562	Inositol phosphate metabolism	1/88	0.831363593	1	0		1	8281597
rcu00010	Glycolysis / Gluconeogenesis	2/88	0.831363593	2	1	8288718	1	8277848
rcu00710	Carbon fixation in photosynthetic organisms	1/88	0.833610827	1	1	8288718	0	
rcu01200	Carbon metabolism	4/88	0.833610827	4	3	8266552/8267717/8288718	1	8288434
rcu04145	Phagosome	1/88	0.833610827	1	1	8284533	0	
rcu00620	Pyruvate metabolism	1/88	0.863066437	1	1	8288718	0	
rcu04146	Peroxisome	1/88	0.863066437	1	1	8277590	0	
rcu00040	Pentose and glucuronate interconversions	1/88	0.872474888	1	1	107260723	0	
rcu03010	Ribosome	1/88	0.99531331	1	0		1	8286431
