# Supplementary material for: Lupeol Accumulation Correlates with Auxin in the Epidermis of Castor
Source: Molecules. 2021 May 17;26(10):2978. doi: 10.3390/molecules26102978 (PMC8156332; doi:10.3390/molecules26102978)
Supplement: Supplementary file 1 [file molecules-26-02978-s001.zip › Supplemental figures and Tables/Supplemental Figures.pdf]

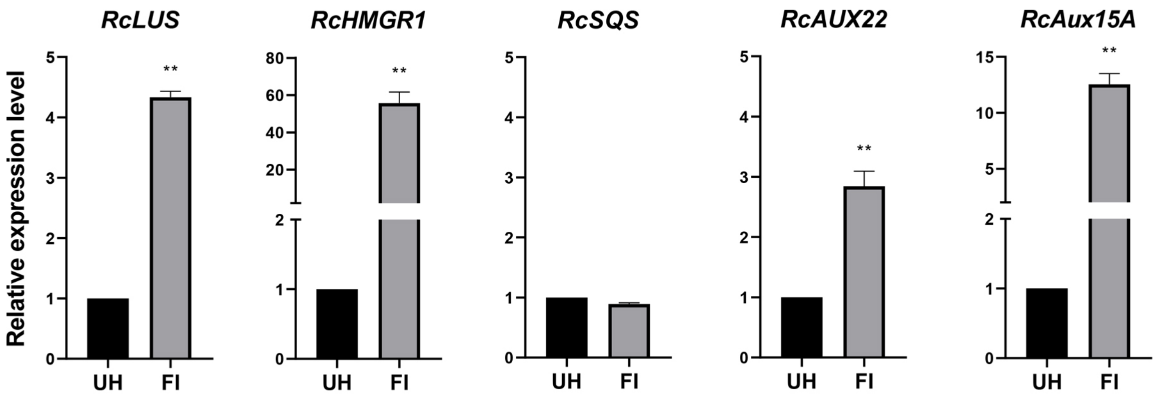

Fig. S1. qRT-PCR validation of transcriptome data. 5 genes were checked including *RcLUS* (LOC8280320), *RcHMGR1* (LOC8258747), *RcSQS* (LOC8284359), *RcAUX22* (LOC8271341) and *RcAux15A* (LOC107262111). The relative expression levels in UH and FI of castor accession line 337 were calculated according to the $2^{-\Delta\Delta Ct}$ method using the castor *RcACT7* (LOC8273907) as internal control.

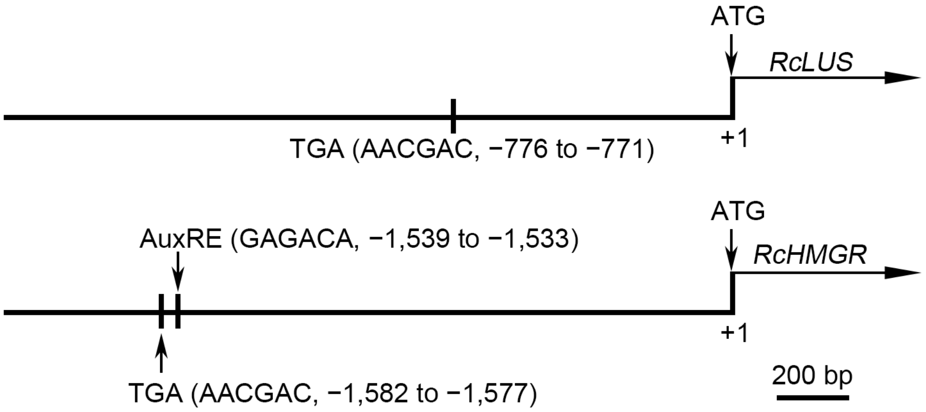

Fig. S2. Schematic representation of the auxin response cis-elements in a 2-kb sequence upstream of the start codon of *RcLUS* and *RcHMGR1*, respectively.
