# Supplementary material for: Lupeol Accumulation Correlates with Auxin in the Epidermis of Castor
Source: Molecules. 2021 May 17;26(10):2978. doi: 10.3390/molecules26102978 (PMC8156332; doi:10.3390/molecules26102978)
Supplement: Supplementary file 1 [file molecules-26-02978-s001.zip › Supplemental figures and Tables/Table S1, S2, S7, S8, S9.pdf]

Table S1. Cuticular wax composition of both upper hypocotyls (UH) and first internodes (FI) of the stem of 337 and 1028 accession lines.

Sample	Total loads	Lupeol	β-amyrin	n-Alkanes	Fatty acids	prim.Alcohols	Aldehydes
1028-UH	59.98 ± 4.73	49.86 ±4.77	1.76 ± 0.15	2.19 ± 0.89	1.79 ± 0.26	2.39 ± 0.29	1.98 ± 0.30
1028-FI	67.67 ± 8.42	57.79 ± 7.33	1.93 ± 0.22	2.10 ± 0.26	1.74 ±0.20	2.41 ± 0.25	1.69 ± 0.44
337-UH	15.75 ± 2.90	8.42 ± 2.21	0.48 + 0.19	0.75 ± 0.21	1.04 ± 0.19	2.97 ± 1.22	2.09 ± 0.80
337-FI	40.29 ± 1.56	28.81 ± 2.80	1.39 ± 0.10	2.06 ± 0.19	1.75 ± 0.41	4.26 ± 1.19	2.03 ± 0.14

Values shown are means±SD (ug/cm2), total wax amounts, and coverage of individual compound classes (n=3-4).

Table S2.Valid data statistics of RNA sequencing.

Sample	Clean_reads	Clean_bases	Total_mapped reads	Q20	Q30	GC (%)
Rc337FI-1	46177990	6.93G	43231216 (93.62%)	97.92	93.96	42.53
Rc337FI-2	50748820	7.61G	48189091 (94.96%)	98.11	94.25	42.18
Rc337FI-3	45015810	6.75G	42246327 (93.85%)	97.93	93.97	41.96
Rc337UH-1	50653290	7.6G	47523981 (93.82%)	98.26	94.72	44.03
Rc337UH-2	43073810	6.46G	40001572 (92.87%)	98.22	94.66	43.69
Rc337UH-3	53869604	8.08G	51105487 (94.87%)	98.42	95	42.16

GC, GC contents of the reads. Q20, the base quality score (Q score) was no less than20. Q30, the base quality score was no less than 30.

Table S7. List of candidate genes of lupeol biosynthesis related to MVA pathway in castor. FC means fold change (First Internode vs Upper Hypocotyl).

Enzymes	Gene_id	log2 FC	padj	Gene description	Gene name
AACT	LOC8276788	-0.081624572	0.943766778	acetyl-CoA acetyltransferase	*RcAACT*
	LOC8289695	-0.332486273	0.755605281	acetyl-CoA acetyltransferase	*RcAACT-Like1*
	LOC8280756	0.252134712	0.811059881	acetyl-CoA acetyltransferase	*RcAACT-Like2*
HMGS	LOC8265393	0.707035306	0.564144259	hydroxymethylglutaryl-CoA synthase	*RcHMGS*
HMGR	LOC8258747	6.167913341	1.16E-29	3-hydroxy-3-methylglutaryl-coenzyme A reductase 1	*RcHMGR*
	LOC8273668	-0.016668463	0.990069483	3-hydroxy-3-methylglutaryl-coenzyme A reductase 1	*RcHMGR-Like1*
	LOC8285370	-0.563552577	0.689726118	3-hydroxy-3-methylglutaryl-coenzyme A reductase 1	*RcHMGR-Like2*
MK	LOC8285549	-0.158134971	0.873940597	mevalonate kinase	*RcMK*
	LOC8260664	0.205074613	0.851160797	mevalonate kinase	*RcMK-Like1*
MVP	LOC8270372	0.219059791	0.77954419	phosphomevalonate kinase	*RcMVP*
PMD	LOC8262804	-0.173180266	0.875481929	diphosphomevalonate decarboxylase MVD	*RcPMD*
IDI	LOC8274290	-0.759183296	0.247657107	isopentenyl-diphosphate Delta-isomerase I	*RcIDI*
FPS	LOC8262022	0.576394892	0.64399742	farnesyl pyrophosphate synthase 1	*RcFPS*
	LOC8286887	0.478288603	0.755902771	farnesyl pyrophosphate synthase 1	*RcFPS-Like1*
SQS	LOC8284359	0.496380317	0.70942241	squalene synthase	*RcSQS*

**SQE**	LOC8267674	0.421942541	0.717454632	squalene monooxygenase	*RcSQE*
	LOC8265245	-0.326374694	0.776750862	squalene monooxygenase	*RcSQE-Like1*
	LOC8281344	-0.8384116	0.946068325	squalene monooxygenase	*RcSQE-Like2*
	LOC112535318	-0.165418032	0.949346403	squalene monooxygenase	*RcSQE-Like3*
**LUS**	LOC8280320	2.877440334	0.009356628	lupeol synthase	*RcLUS*
	LOC8283084	1.674874711	0.588559215	lupeol synthase	*RcLUS-like1*

Table S8. List of candidate genes of lupeol biosynthesis related to Methylerythritol 4-phosphate (MEP) pathway in castor. FC means fold change (First Internode vs Upper Hypocotyl).

Enzymes	Gene_id	log2 FC	padj	Gene description
DXS	LOC8262363	0.385930715	0.643213288	1-deoxy-D-xylulose-5-phosphate synthase
	LOC8277645	0.92212388	0.717297417	1-deoxy-D-xylulose-5-phosphate synthase
	LOC8284855	0.662339731	0.515170739	1-deoxy-D-xylulose-5-phosphate synthase
	LOC8277493	1.78248988	0.120339421	1-deoxy-D-xylulose-5-phosphate synthase
DXR	LOC8289006	-0.531555526	0.731601492	1-deoxy-D-xylulose 5-phosphate reductoisomerase
CMS/MCT	LOC8262623	-0.917767175	0.365148894	2-C-methyl-D-erythritol 4-phosphate cytidylyltransferase
CMK	LOC8268775	-0.321579311	0.807367198	4-diphosphocytidyl-2-C-methyl-D-erythritol kinase
MCS(MDS)	LOC8287434	-0.545608358	0.666101005	2-C-methyl-D-erythritol 2,4-cyclodiphosphate synthase
HDS	LOC8264075	0.180030545	0.8800589	4-hydroxy-3-methylbut-2-enyl diphosphate synthase (ferredoxin)
HDR	LOC8279543	0.057630161	0.958950694	4-hydroxy-3-methylbut-2-enyl diphosphate reductase

Table S9. Primers used in this study.

ID	Sequence 5'-3'	Purpose
RcACT7-F	TTGTGCTCAGTGGTGGTTC	qRT-PCR
RcACT7-R	TACTCGCCCTTGGAAATC	qRT-PCR
RcLUS-F	AGTGTGCGTCATTTATGTGC	qRT-PCR
RcLUS-R	GCCAACGAGAGAATAGAGGC	qRT-PCR
RcGH3-F	CTCTTACACCCTCCTACCAAATA	qRT-PCR
RcGH3-R	CTTCTTCTTCTTCTCAACCTCCT	qRT-PCR
RcAUX22-F	GCAATAGTGCCTCCTCCAC	qRT-PCR
RcAUX22-R	GTTTCAAGCCTTTCTTTCTCAT	qRT-PCR
RcAux15A-F	CCGAAAGGCCATGTTGC	qRT-PCR
RcAux15A-R	ACCACCCATTGGATGATTAA	qRT-PCR
RcHMGR1-F	CCTATGAAATCTACCTCCTCCG	qRT-PCR
RcHMGR1-R	GCAGAAAGTAAGCAACCGAAA	qRT-PCR
RcSQS-F	AGCGAGGTTCTGGTGGG	qRT-PCR
RcSQS-R	TGGGTAGTGTTGGAGCGATTT	qRT-PCR
